# Conversion hepatectomy and survival in initially unresectable hepatocellular carcinoma: a systematic review and meta-analysis with quantitative bias analysis

**DOI:** 10.1186/s12957-026-04498-x

**Published:** 2026-07-16

**Authors:** Wenguang Zhao, Xialin Zhang, Ao Zhong, Chenxi Jiang, Ran Cui, Xinlin Yu, Sheng Lin

**Affiliations:** 1 Department of Oncology, Affiliated Hospital of Southwest Medical University, No. 25 Taiping Street, Jiangyang District, Luzhou, Sichuan 646000 China; 2 Department of Respiratory and Critical Care Medicine, First People’s Hospital of Neijiang, Neijiang, Sichuan 641000 China

**Keywords:** Hepatocellular carcinoma, Conversion therapy, Hepatectomy, Surgical oncology, Meta-analysis, Quantitative bias analysis, E-value, Immortal-time bias, Publication bias, Systematic review

## Abstract

**Background:**

Conversion hepatectomy has become feasible for selected patients with initially unresectable hepatocellular carcinoma (HCC), with comparative studies reporting longer survival after surgery. We performed a systematic review, meta-analysis, and quantitative bias analysis (QBA) to assess whether residual selection and time-dependent treatment bias could account for the reported benefit.

**Methods:**

We systematically reviewed comparative studies of conversion or salvage hepatectomy versus non-surgical management in initially unresectable HCC and pooled endpoint-specific HRs for OS and PFS/EFS using random-effects models. For each estimate, we extracted the time origin, exposure definition, and adjustment model. Risk of bias and certainty of evidence were assessed using ROBINS-I and GRADE. Robustness to unmeasured confounding, immortal time bias, and small-study effects was evaluated using E-values, tipping-point analysis, probabilistic QBA, timing-bias scenario analyses, and funnel-plot diagnostics.

**Results:**

Fourteen studies contributed 24 h: 14 for OS and 10 for PFS/EFS. Hepatectomy was associated with lower hazards of death (HR 0.33, 95% CI 0.22–0.48) and progression or event (HR 0.41, 95% CI 0.28–0.59), although heterogeneity was substantial and both prediction intervals crossed 1.0. E-values suggested robustness to moderate unmeasured confounding, whereas extreme surgical-candidacy confounding attenuated the median bias-adjusted HR to 0.82 for OS and 1.02 for PFS/EFS. Immortal-time bias scenarios did not fully account for the observed associations. OS small-study-effect diagnostics suggested funnel-plot asymmetry, although sample-size-based Peters-type regression was not significant. Certainty of evidence was very low for both endpoints.

**Conclusions:**

Conversion hepatectomy was associated with better observed survival outcomes in selected patients with initially unresectable HCC after conversion-intent therapy. The association was robust to moderate, but not extreme, assumptions about unmeasured surgical-candidacy confounding. These findings should be interpreted as a composite post-conversion association reflecting surgical clearance, treatment response, favorable tumor biology, and patient selection rather than as the isolated causal effect of hepatectomy. Future studies should report surgical eligibility landmarks, time zero, time to hepatectomy, and reasons for non-resection to clarify the independent contribution of surgery within conversion pathways.

**Trial registration:**

PROSPERO CRD420261382843 (https://www.crd.york.ac.uk/prospero/display_record.php?RecordID=1382843).

**Supplementary Information:**

The online version contains supplementary material available at https://doi.org/10.1186/s12957-026-04498-x.

## Background

Initially unresectable hepatocellular carcinoma (HCC) is associated with poor prognosis and limited access to curative-intent treatment [1]. Advances in systemic therapy, including atezolizumab plus bevacizumab in IMbrave150 and tremelimumab plus durvalumab in HIMALAYA, have improved survival in unresectable disease and expanded the therapeutic landscape [2, 3]. Together with locoregional and combination strategies, these advances have enabled selected initially inoperable patients to achieve sufficient tumor regression and liver-function reserve for reconsideration of hepatectomy, forming the clinical basis for conversion therapy [4].

Conversion hepatectomy is increasingly considered within multidisciplinary management for selected patients. Cohort studies comparing post-conversion hepatectomy with non-surgical management generally report better survival in the surgical group. The key question is therefore not only whether conversion-intent therapy can achieve tumor response or resectability, but whether proceeding to hepatectomy provides additional survival benefit once resection becomes feasible.

Previous reviews of conversion therapy in HCC have primarily focused on feasibility and antitumor activity, including objective response, conversion-to-surgery rate, resection rate, and safety [5, 6]. The only survival-focused meta-analysis was limited to patients with complete response, a narrow setting in which conclusions varied by response definition and comparator [7]. Survival associated with proceeding to hepatectomy in the broader conversion-therapy population has not been systematically evaluated. Comparative estimates are also vulnerable to selection and timing-related bias because hepatectomy is performed only after treatment initiation. Surgical patients are therefore selected by survival to surgery, treatment response, liver-function reserve, and technical resectability [8]. A hazard ratio favoring hepatectomy may capture both residual tumor removal and the favorable biology and clinical profile of patients selected for surgery, while delayed exposure classification may introduce immortal-time bias if preoperative follow-up time is assigned to the surgical group [9]. Prior evidence syntheses have not formally evaluated whether residual surgical-candidacy confounding and immortal-time bias could plausibly account for the reported hepatectomy-comparison survival associations [5–7].

We conducted a systematic review and random-effects meta-analysis of comparative survival studies evaluating conversion or salvage hepatectomy in initially unresectable HCC, and applied quantitative bias analysis to assess whether residual surgical selection and surgery-related timing bias could plausibly account for the pooled associations.

## Methods

### Study design and protocol

The protocol was prospectively registered in PROSPERO (CRD420261382843). This systematic review was conducted in accordance with the Cochrane Handbook for Systematic Reviews of Interventions and reported according to the PRISMA 2020 statement [10, 11]. The literature search was updated during manuscript revision without changes to the prespecified PICO framework. No substantive amendments to the registered protocol were made. The complete database-specific search strategies are provided in Additional file 1 (Table S1).

### Eligibility criteria

Eligible studies were full-text original clinical reports enrolling adults with initially unresectable, initially inoperable, or non-upfront-resectable hepatocellular carcinoma who received conversion-intent treatment and were subsequently compared according to hepatectomy versus non-hepatectomy management. Eligible reports had to provide extractable comparative time-to-event HRs with 95% CIs for OS, PFS, EFS, DFS, or RFS, provided that the estimate explicitly isolated hepatectomy status.

Reports were excluded if they were limited to initially resectable neoadjuvant populations, postoperative adjuvant-only treatment, transplantation-only downstaging, ablation-only conversion, non-original articles, case reports, duplicate cohorts, prognostic-factor-only analyses, or regimen-comparison analyses that did not isolate hepatectomy status. Locoregional, hepatic arterial infusion, systemic, and multimodal conversion-intent regimens were eligible when the report provided an extractable comparative HR for hepatectomy versus non-hepatectomy management. Initially unresectable disease was accepted according to the original study definition, protocol designation, or multidisciplinary treatment context [12]. We did not retrospectively impose a uniform technical unresectability definition across heterogeneous published cohorts.

### Study selection and data extraction

Study screening, data extraction, estimand classification, and risk-of-bias assessment were performed independently by two reviewers using standardized, pilot-tested extraction forms to ensure consistency across the large volume of records. Disagreements were resolved by consensus or independent third-reviewer adjudication. For reports meeting eligibility criteria, we extracted study design, treatment context, analytic cohort size, comparison definition, survival endpoint, HR, 95% CI, time origin, exposure definition, HR source, adjustment method, adjustment status, covariates included in adjusted models when reported, and source location.

For each extracted HR, we recorded whether the estimate was directly reported, model-derived, Kaplan–Meier-derived, adjusted, unadjusted, landmark-based, or time-dependent. HRs were not entered into synthesis unless the comparison isolated hepatectomy versus non-hepatectomy management. When the original report did not provide a single unambiguous time origin, the estimate was retained if the comparison was clinically interpretable, and time origin was coded as unclear for risk-of-bias interpretation and supplementary diagnostics. HRs for age, alpha-fetoprotein, portal vein tumor thrombus, tumor burden, radiologic response, pathological response, postoperative recurrence predictors, postoperative adjuvant therapy, and treatment-regimen comparisons were excluded unless hepatectomy status was explicitly isolated. An extraction table was maintained for all extracted comparative survival estimates (Additional file 1: Table S3).

### Target contrast and HR eligibility

The target contrast was the endpoint-specific survival association comparing patients who underwent conversion hepatectomy with those managed without hepatectomy after conversion-intent therapy. We interpreted this contrast as a composite prognostic-therapeutic association rather than as the isolated causal effect of hepatectomy. We explicitly conceptualize hepatectomy not solely as a therapeutic intervention, but also as a surrogate marker of favorable tumor biology, successful conversion, and preserved liver function.

Eligible estimates were classified according to the relation between follow-up, surgery assignment, and conversion eligibility. Treatment-initiation-anchored estimates began follow-up at conversion therapy initiation and classified exposure by later receipt of hepatectomy. Conditional estimates began follow-up after response, conversion eligibility, radiologic complete response, or another post-treatment landmark. Time-dependent estimates modeled hepatectomy as a time-updating exposure. This classification was used to describe the timing structure of the extracted estimates and to inform risk-of-bias interpretation. Target-trial principles were used conceptually to evaluate whether eligibility, treatment assignment, time zero, follow-up, and outcome definition were aligned [13, 14].

### ROBINS-I-informed risk-of-bias assessment

Risk of bias was summarized using ROBINS-I-informed domains focused on confounding, participant selection, intervention classification, post-treatment timing or immortal-time concerns, endpoint coherence, and overall interpretability. The assessment was adapted to the review question because hepatectomy was generally assigned after conversion-intent therapy rather than at treatment initiation.

For each endpoint-specific estimate, we recorded timing and design features relevant to bias interpretation, including clarity of time origin, timing of surgery assignment, immortal-time-bias concern, adjustment for baseline surgical candidacy, adjustment for treatment response, use of landmark analysis, and use of time-dependent Cox modeling. Endpoint-level judgments and support for judgment notes are provided in Additional file.

### Certainty of evidence assessment

We assessed the certainty of the body of evidence for each synthesized outcome using an adapted GRADE framework. Certainty was judged separately for OS and PFS/EFS, considering study design, risk of bias, inconsistency, indirectness, imprecision, publication bias, and potential upgrading factors. ROBINS-I-informed domain judgments were used to inform the risk-of-bias domain, while heterogeneity statistics, prediction intervals, endpoint definitions, time-origin variability, and QBA findings informed the remaining domains. Because all included estimates came from non-randomized comparisons in which hepatectomy was usually assigned after treatment initiation, the GRADE assessment was specifically adapted to evaluate the certainty of these comparative prognostic and therapeutic associations.

### Statistical synthesis of survival HRs

Endpoint-specific HRs were synthesized on the log-HR scale. For each HR, the standard error was calculated from the reported 95% CI as SE = [log(upper limit) − log(lower limit)] / 3.92. Separate random-effects meta-analyses were performed for OS and PFS/EFS using restricted maximum likelihood estimation. Pooled estimates were back-transformed and reported as HRs with 95% CIs. Between-study heterogeneity was summarized using I² and τ², and 95% prediction intervals were calculated to describe the expected range of effects in comparable future settings. PFS and EFS were grouped pragmatically because both capture progression- or event-related milestones prior to death; original endpoint labels were retained in study-level tables.

### E-value analysis

For protective associations, the inverse HR was used before applying the standard E-value formula. Confidence-limit E-values were calculated using the confidence bound closest to the null [15, 16].

E-values were interpreted as the minimum strength of association that an unmeasured confounder would need to have with both receipt of hepatectomy and the survival outcome, conditional on measured covariates, to move the observed association to the null. This framework specifically addresses residual confounding rather than timing-related mechanisms.

### Deterministic tipping-point QBA

Deterministic tipping-point quantitative bias analysis was performed using the endpoint-specific pooled HRs from the random-effects syntheses as observed inputs, applying the standard maximum bias-factor approach for unmeasured confounding [17]. Full details of the bias-factor calculation and the clinical plausibility grid are provided in Additional file 1 (Methods S3).

### Probabilistic QBA

Probabilistic QBA assigned prespecified log-normal distributions to the exposure-confounder and confounder-outcome associations under moderate, strong, and extreme surgical-candidacy confounding scenarios. Full distributional assumptions and Monte Carlo settings are reported in Additional file.

### Aggregate-data timing-bias scenario analysis

Because individual patient-level time-to-surgery data were unavailable across studies, immortal-time bias could not be formally corrected using a unified time-dependent model [18, 19]. We therefore performed an aggregate scenario analysis to examine how the pooled estimates would change under different assumptions about the time from treatment initiation to surgery and the median survival of the non-surgical comparator group. The calculation details are provided in Additional file.

### Sensitivity and influence analyses

Regimen-based subgroup meta-analysis was not planned because treatment regimens were heterogeneous and sparsely represented within comparable hepatectomy contrasts. Prespecified descriptive diagnostics included leave-one-out analysis, exclusion of Kaplan–Meier-derived estimates, exclusion of extreme HRs, adjusted or time-dependent estimates only, directly reported HRs only where applicable, and influence diagnostics. Because the OS synthesis included 14 estimates, we assessed small-study effects and reporting-bias risk for OS using a contour-enhanced funnel plot, SE-based Egger regression, sample-size-based Peters-type regression, and rank-correlation testing. The PFS/EFS synthesis included 10 estimates and combined related but non-identical endpoints; therefore, its funnel plot and regression diagnostics were treated as descriptive only. These analyses were used to inform GRADE publication-bias judgments rather than to redefine the main synthesis.

### Software and reproducibility

Analyses were performed in R version 4.5.3 using prespecified analysis code. Random-effects meta-analysis, prediction intervals, leave-one-out analysis, influence diagnostics, and small-study-effect tests were implemented with the metafor package [20]. E-value calculations, deterministic tipping-point QBA, probabilistic QBA, timing-bias scenario analyses, and funnel-plot diagnostics were applied to the extracted comparative HR dataset. Package versions, session information, extraction tables, and analysis code were archived to support reproducibility [21].

## Results

### Study selection and included studies

**Fig. 1:**
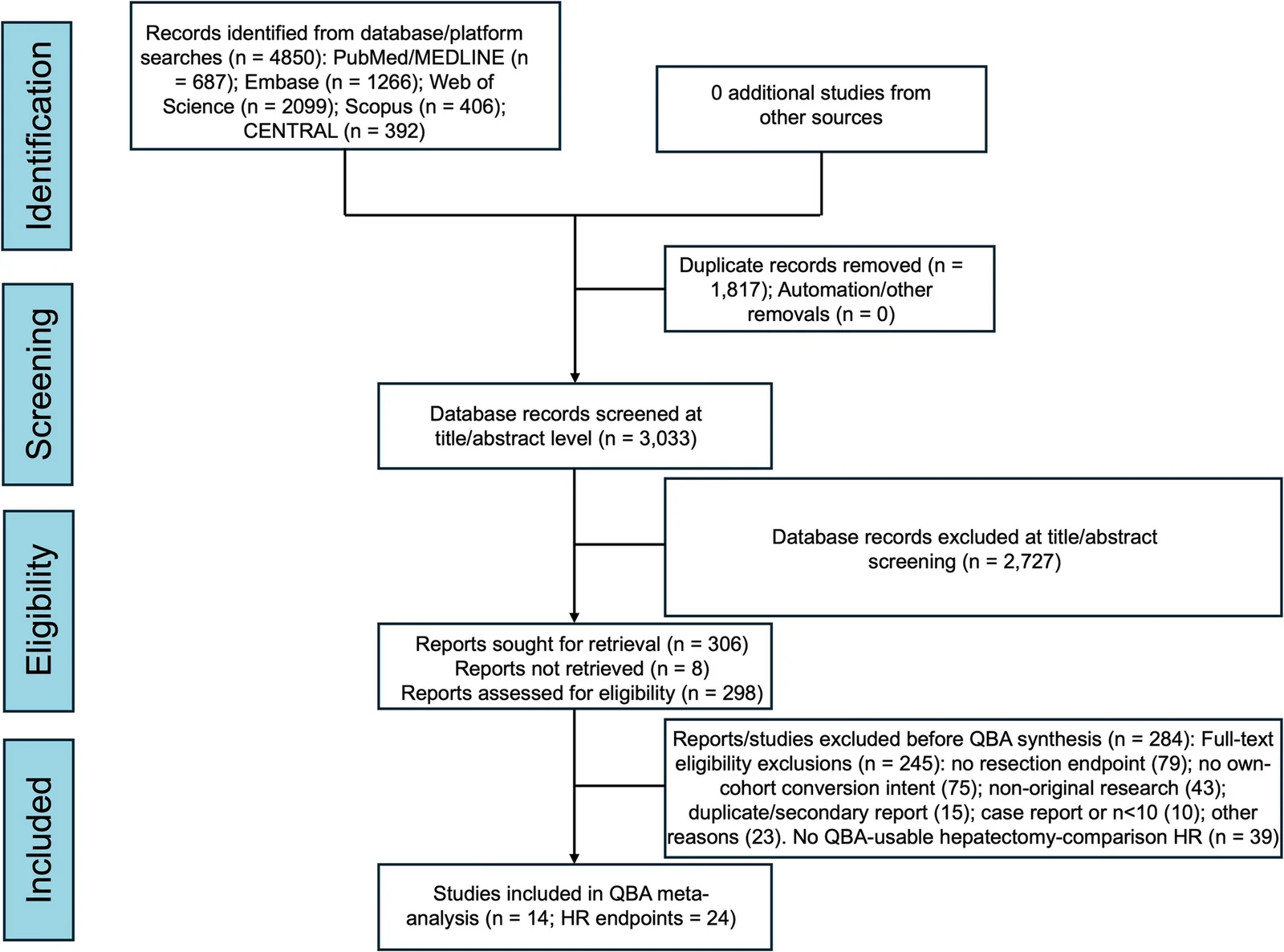
PRISMA 2020 flow diagram for selection of comparative survival studies. The diagram shows database and platform records identified, duplicate removal, title/abstract screening, full-text retrieval, full-text eligibility assessment, reports excluded before QBA synthesis, and final inclusion of 14 comparative studies contributing 24 endpoint-specific HRs

Fourteen comparative survival studies met the eligibility criteria and contributed 24 endpoint-specific HRs comparing hepatectomy with non-hepatectomy management, including 14 OS estimates and 10 PFS/EFS estimates (Fig. 1).

### Study and estimand characteristics

The 14 studies included analytic cohorts ranging from 36 to 1262 patients. Treatment contexts included tyrosine kinase inhibitor- and immune checkpoint inhibitor-based combinations, HAIC-based strategies, TACE/HAIC-containing multimodal therapy, and real-world immune-based conversion regimens. Comparator definitions generally contrasted patients undergoing conversion or salvage hepatectomy with patients managed without hepatectomy after conversion-intent therapy. Time origin varied across studies. Some estimates were anchored at treatment initiation, some were conditional on response or conversion eligibility, and one study used a time-dependent Cox framework. Study characteristics are summarized in Table 1, with full study details in Additional file 1: Table S2. Endpoint-specific HRs with corresponding E-values are listed in Table 2, and the complete extraction table is provided in Additional file 1: Table S3.

**Table 1:** Characteristics of included comparative studies and extracted hepatectomy-comparison estimates

Study	Cohort *n*	Regimen class	Comparison	Endpoints	HR source/model
Chi QY 2025 [22]	74	lenvatinib + PD-1 inhibitor + TACE + HAIC	Surgery vs. no surgery	OS, PFS	MVA Cox
Gao W 2025 [23]	36	donafenib + sintilimab + HAIC	Surgery vs. no surgery	EFS, OS	MVA Cox
Goto Y 2025 [24]	153	HAIC	hepatectomy/conversion surgery vs. non-hepatectomy	OS, PFS	KM-derived
Li X 2023 [25]	94	lenvatinib + PD-1 inhibitor + TACE	Surgery vs. no surgery	PFS, OS	MVA Cox
Lin S 2025 [26]	301	TACE + HAIC	hepatectomy/conversion surgery vs. non-hepatectomy	OS, PFS	TD-Cox
Lu YC 2024 [27]	51	HAIC	Surgery vs. no surgery	OS, PFS	KM-derived
Pang B 2024 [28]	62	PD-1 inhibitor + TACE + HAIC	Surgery vs. no surgery	PFS, OS	KM-derived
Piao M 2025 [29]	613	ICI-based combination therapy	Surgery vs. no surgery	PFS, OS	KM-derived/unadjusted
Wang L 2024 [30]	93	LRT + TKI + anti-PD-1	Surgery vs. no surgery	OS, PFS	KM-derived
Xue Z 2025 [31]	79	sorafenib + PD-1 inhibitor	Surgery vs. no surgery	OS	MVA Cox
Yang DL 2025 [32]	1262	immune-based conversion therapy	Hepatectomy vs. other downstream treatment	OS	Unadjusted Cox; rCR-anchored
Zhu XD 2023 [33]	101	TKI + anti-PD-1 antibody therapy	hepatectomy/conversion surgery vs. non-hepatectomy	OS	MVA Cox
Sun Z 2026 [34]	67	ICI + TKI	Surgery vs. no surgery	OS, PFS	Univariate/MVA Cox
Wu JY 2024 [35]	144	TACE + lenvatinib + anti-PD-1	Salvage surgery vs. non-salvage surgery	OS	KM-derived approximate

Trial-setting labels describe the source cohort context and do not imply that hepatectomy itself was randomized; the surgery contrast remained non-randomized and was assigned after treatment initiation

**Table 2:** Endpoint-specific hepatectomy-comparison HRs and E-values

Study	Endpoint	HR (95% CI)	Model	Adjustment	E-value
Chi QY 2025 [22]	OS	0.19 (0.06–0.66)	MVA Cox	Adjusted	9.78
Gao W 2025 [23]	OS	0.24 (0.06–0.97)	MVA Cox	Adjusted	7.8
Li X 2023 [25]	OS	0.15 (0.04–0.55)	MVA Cox	Adjusted	13.09
Lin S 2025 [26]	OS	0.17 (0.07–0.40)	TD-Cox	Adjusted	11.24
Xue Z 2025 [31]	OS	0.39 (0.18–0.85)	MVA Cox	Adjusted	4.59
Zhu XD 2023 [33]	OS	0.05 (0.01–0.36)	MVA Cox	Adjusted	39.49
Goto Y 2025 [24]	OS	0.44 (0.27–0.72)	KM-derived	Unadjusted	3.93
Lu YC 2024 [27]	OS	0.12 (0.02–0.63)	KM-derived	Unadjusted	16.15
Pang B 2024 [28]	OS	0.52 (0.28–0.99)	KM-derived	Unadjusted	3.23
Piao M 2025 [29]	OS	0.26 (0.14–0.46)	KM-derived	Unadjusted	7.15
Wang L 2024 [30]	OS	1.24 (0.78–1.97)	KM-derived	Unadjusted	1.78
Yang DL 2025 [32]	OS	0.36 (0.18–0.74)	Unadjusted Cox; rCR-anchored	Unadjusted	5
Chi QY 2025 [22]	PFS/EFS	0.34 (0.12–0.97)	MVA Cox	Adjusted	5.4
Gao W 2025 [23]	PFS/EFS	0.10 (0.02–0.51)	MVA Cox	Adjusted	19.49
Li X 2023 [25]	PFS/EFS	0.25 (0.10–0.62)	MVA Cox	Adjusted	7.53
Lin S 2025 [26]	PFS/EFS	0.37 (0.22–0.61)	TD-Cox	Adjusted	4.85
Goto Y 2025 [24]	PFS/EFS	0.42 (0.25–0.70)	KM-derived	Unadjusted	4.19
Lu YC 2024 [27]	PFS/EFS	0.20 (0.07–0.58)	KM-derived	Unadjusted	9.47
Pang B 2024 [28]	PFS/EFS	0.44 (0.24–0.81)	KM-derived	Unadjusted	3.93
Piao M 2025 [29]	PFS/EFS	0.39 (0.24–0.63)	KM-derived	Unadjusted	4.57
Wang L 2024 [30]	PFS/EFS	1.25 (0.79–1.98)	KM-derived	Unadjusted	1.82
Sun Z 2026 [34]	OS	0.56 (0.25–1.43)	Univariate Cox	Unadjusted	3.00
Wu JY 2024 [35]	OS	0.25 (0.09–0.68)	KM-derived approximate	Unadjusted; approximate	7.46
Sun Z 2026 [34]	PFS/EFS	0.46 (0.23–0.91)	MVA Cox	Adjusted	3.82

*HR*  Hazard ratio, *CI*  Confidence interval, *KM*  Kaplan-Meier, *MVA*  Multivariable adjustment, *TD-Cox*  Time-dependent Cox. E-values calibrate the strength of unmeasured confounding required to move an observed association to the null. Confidence-limit E-values and full extraction fields are provided in Additional file 1: Tables S3 and S6

### Overall survival synthesis

**Fig. 2:**
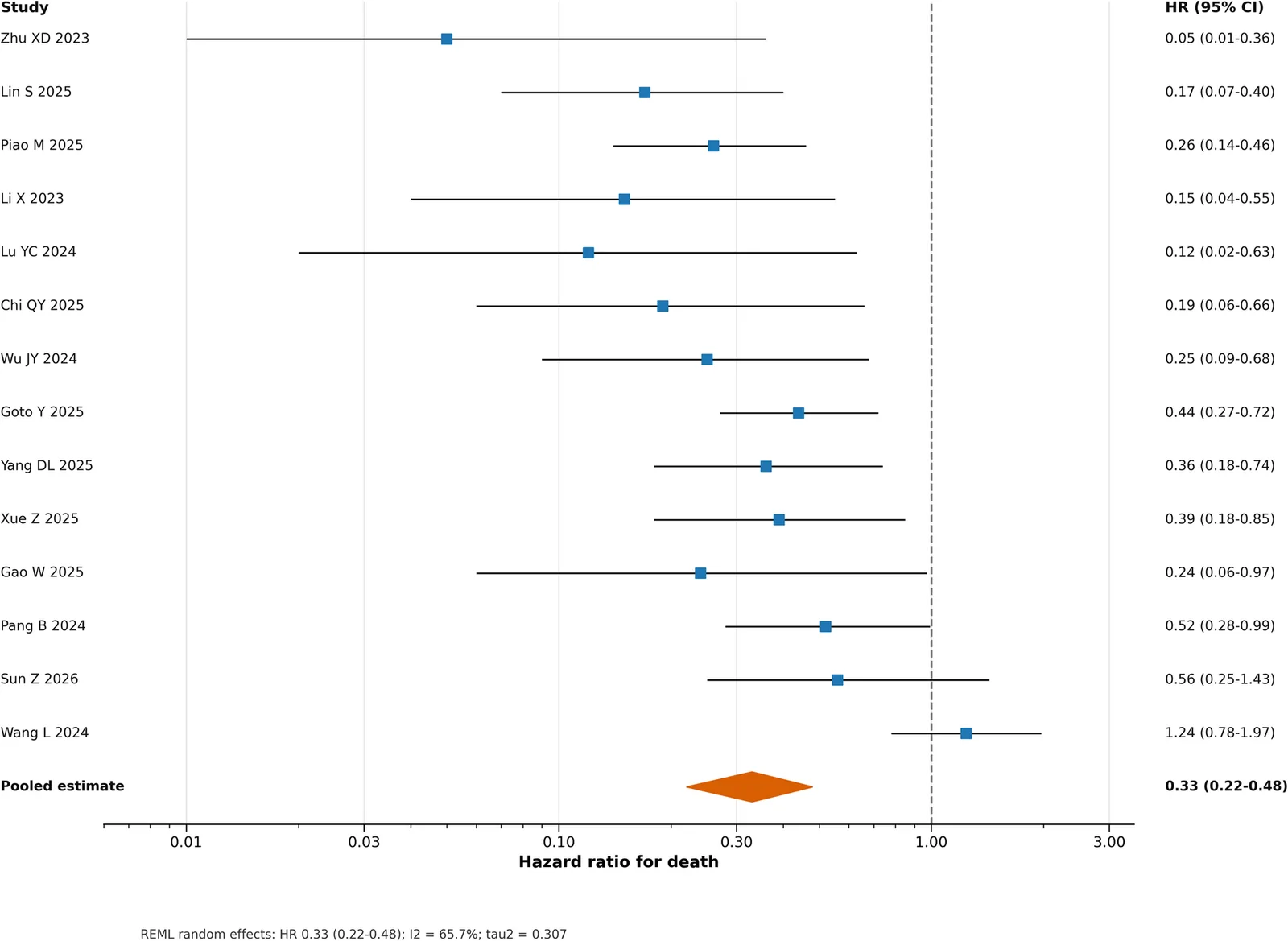
Random-effects synthesis of overall survival HRs. Forest plot of all 14 endpoint-specific OS HRs comparing conversion hepatectomy with non-hepatectomy management. Study-specific HRs and 95% CIs are shown with the random-effects pooled estimate (HR 0.33 [95% CI 0.22–0.48]), heterogeneity statistics, and 95% prediction interval. HR < 1 indicates lower hazard in the hepatectomy group

Fourteen OS-specific HRs were included. Conversion hepatectomy was associated with a lower hazard of death than non-surgical management (HR 0.33, 95% CI 0.22–0.48). Heterogeneity was substantial (I² = 65.7%; τ² = 0.307), and the 95% prediction interval included the null value (0.10–1.04) (Fig. 2 and Table 3).

**Table 3:** Random-effects synthesis and QBA summary of included hepatectomy-comparison HRs

Outcome	HR rows	Pooled HR (95% CI)	Prediction interval	I² (%)	τ²	Pooled E-value	Confidence-limit E-value
Overall survival	14	0.33 (0.22–0.48)	0.10–1.04	65.7	0.307	5.54	3.58
Progression/event-free survival	10	0.41 (0.28–0.59)	0.16–1.05	64.3	0.197	4.34	2.81

All extractable endpoint-specific HRs comparing hepatectomy with non-hepatectomy management were synthesized by endpoint. Random-effects REML estimates are shown. Prediction interval, I², τ², pooled E-value, and confidence-limit E-value are reported

### Progression/event-free survival synthesis

**Fig. 3:**
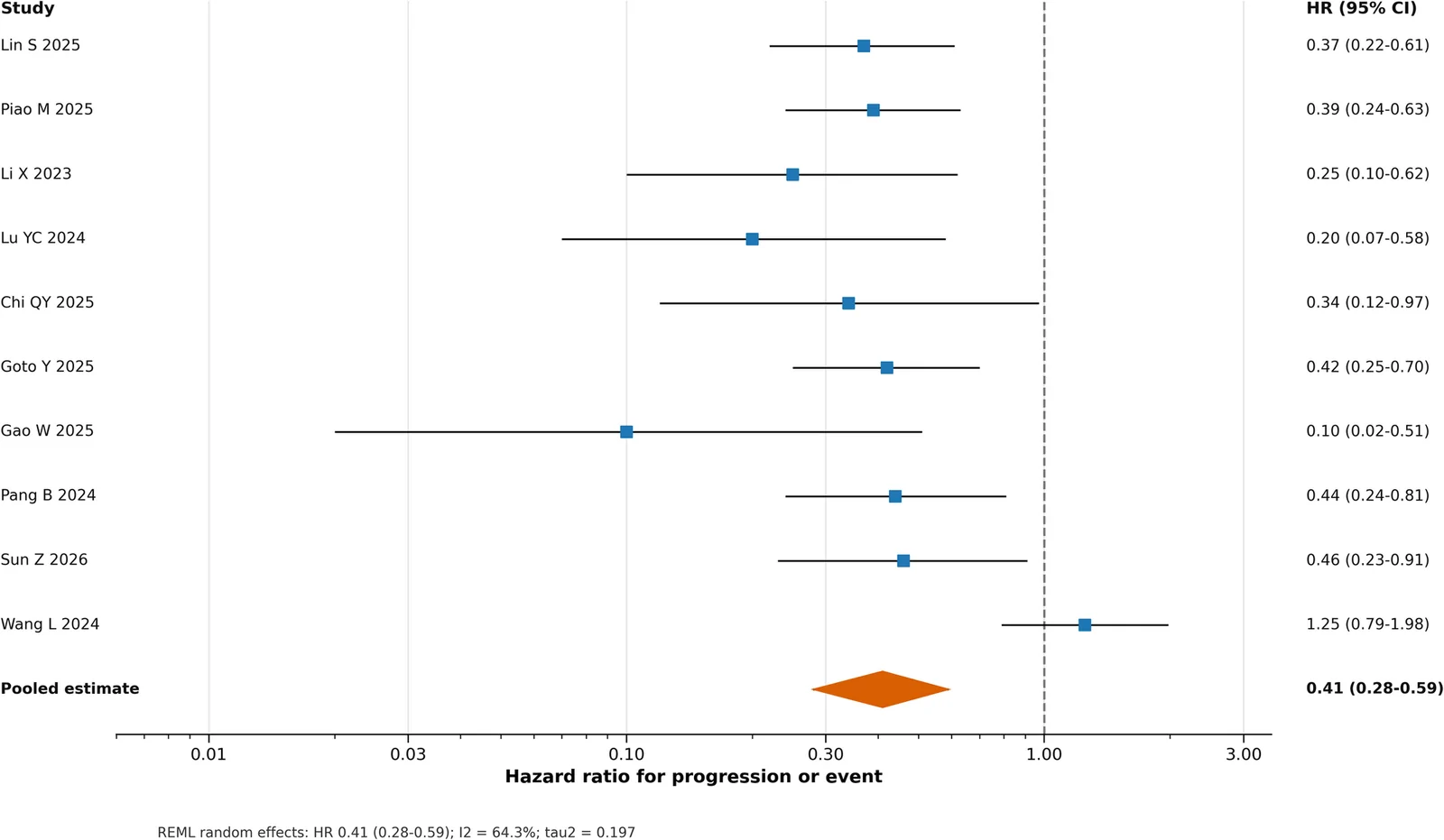
Random-effects synthesis of progression/event-free survival HRs. Forest plot of all 10 endpoint-specific PFS/EFS HRs comparing conversion hepatectomy with non-hepatectomy management. Study-specific HRs and 95% CIs are shown with the random-effects pooled estimate (HR 0.41 [95% CI 0.28–0.59]), heterogeneity statistics, and 95% prediction interval. HR < 1 indicates lower hazard in the hepatectomy group

Ten PFS/EFS-specific HRs were included. Conversion hepatectomy was associated with a lower hazard of progression or qualifying event than non-surgical management (HR 0.41, 95% CI 0.28–0.59). Heterogeneity was substantial (I² = 64.3%; τ² = 0.197), and the 95% prediction interval included the null value (0.16–1.05) (Fig. 3 and Table 3).

### Robustness to unmeasured surgical-candidacy confounding

**Fig. 4:**
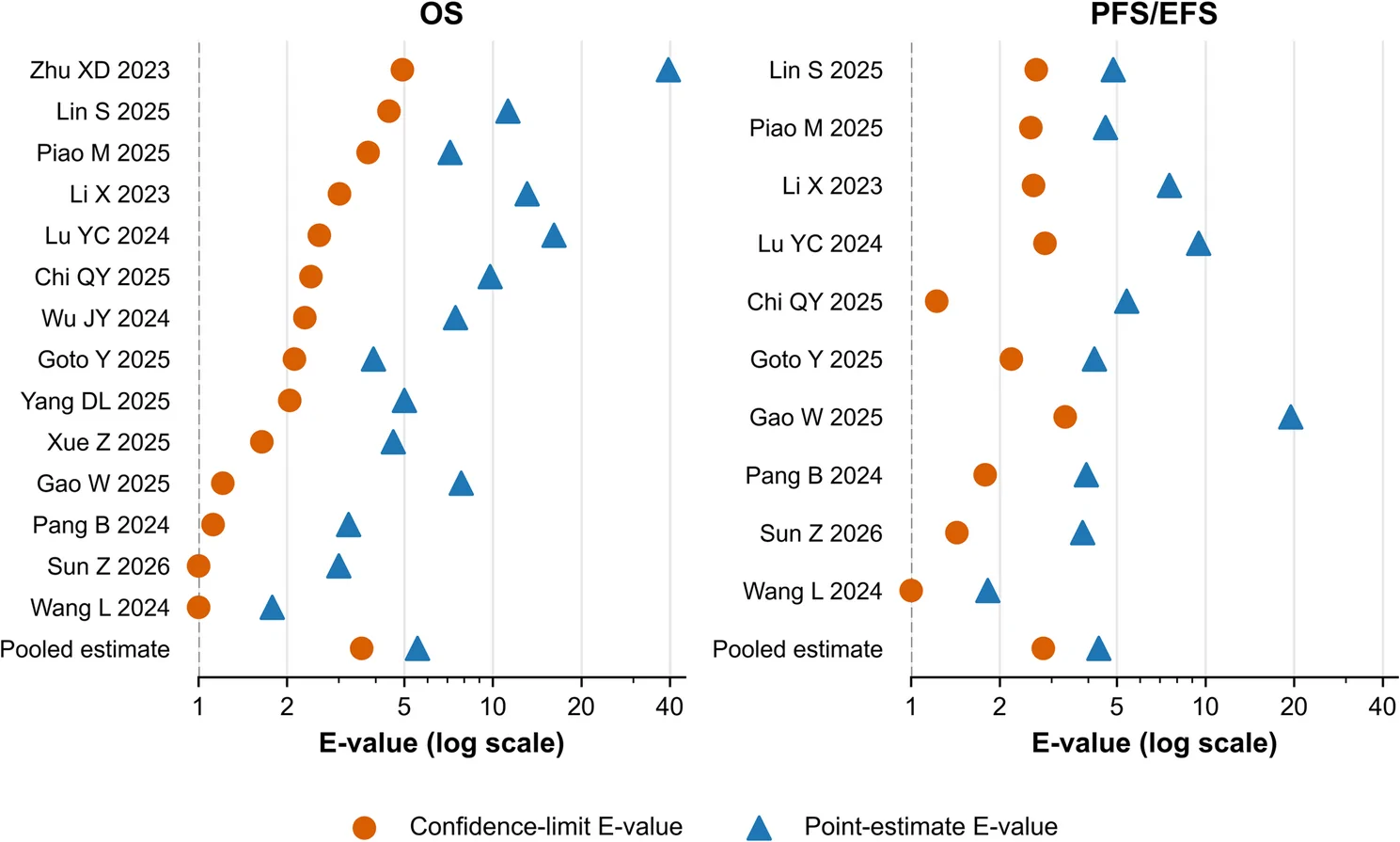
E-values for pooled and study-specific hepatectomy-comparison HRs. Point-estimate and confidence-limit E-values are shown for study-specific and pooled OS and PFS/EFS estimates. Pooled E-values were 5.54 for OS and 4.34 for PFS/EFS; CI-limit E-values were 3.58 and 2.81, respectively. E-values address unmeasured confounding and do not correct immortal-time bias

The pooled OS estimate had a point-estimate E-value of 5.54 and a confidence-limit E-value of 3.58. The corresponding values for PFS/EFS were 4.34 and 2.81, respectively (Fig. 4).

### Bias thresholds under deterministic QBA

**Fig. 5:**
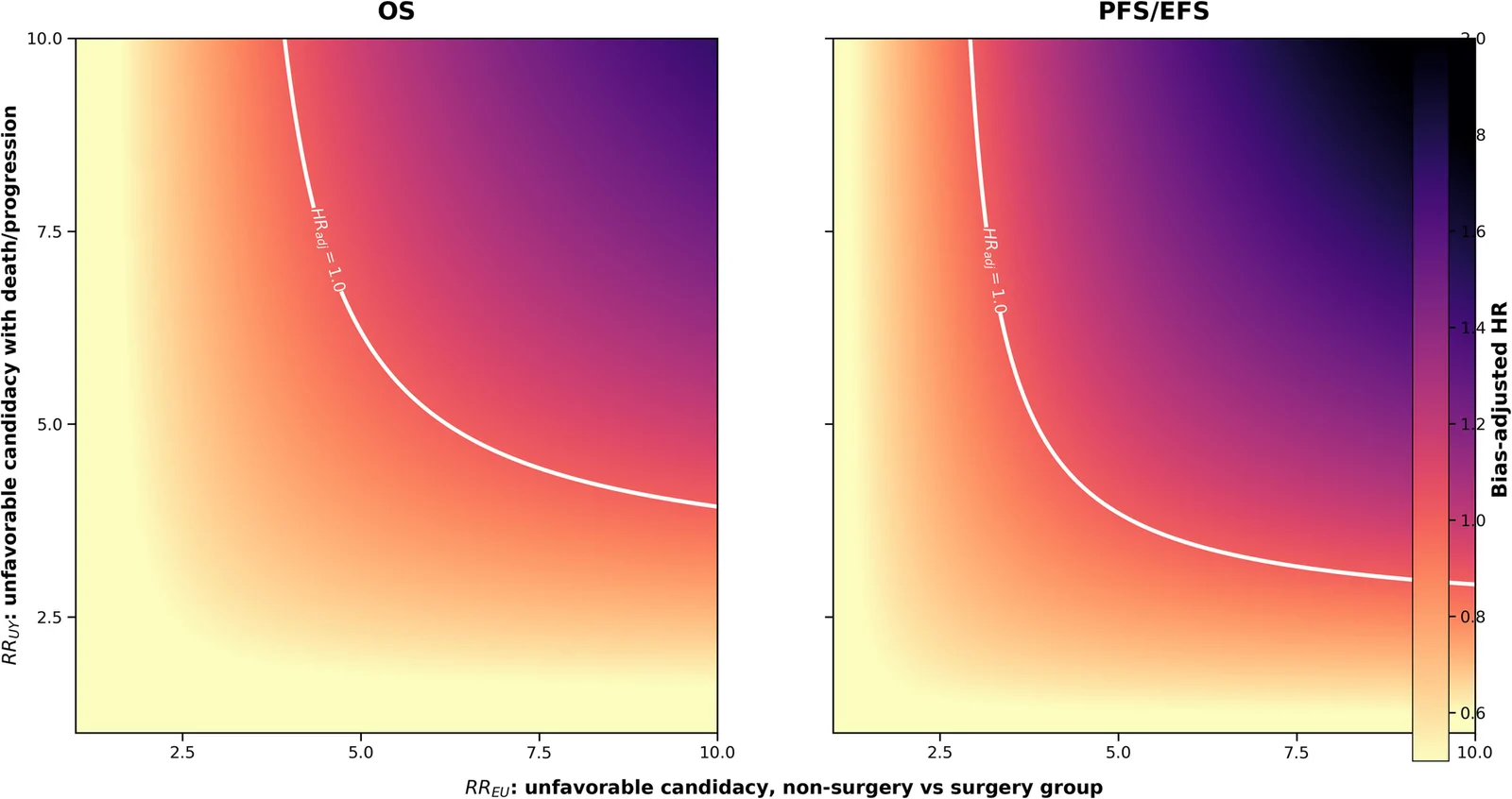
Deterministic QBA for unmeasured surgical-candidacy confounding. Heat maps show bias-adjusted pooled HRs across combinations of exposure-confounder imbalance (RREU) and confounder-outcome association (RRUY), using observed pooled HRs of 0.329 for OS and 0.408 for PFS/EFS. White contours indicate parameter combinations at which the bias-adjusted HR reaches 1

In deterministic tipping-point QBA, modest combinations of surgical-candidacy imbalance and confounder-outcome association did not move the pooled HRs to the null. Stronger joint bias assumptions moved the bias-adjusted HRs toward the null, particularly for PFS/EFS. The rule-out contour identified the combinations at which the adjusted HR reached 1 (Fig. 5).

### Probabilistic bias-adjusted survival associations

Probabilistic QBA shifted the simulated bias-adjusted HR distributions toward the null. For OS, median bias-adjusted HRs were 0.42, 0.55, and 0.82 under moderate, strong, and extreme surgical-candidacy confounding scenarios, respectively; the corresponding probabilities that the adjusted HR remained below 1.0 were 1.000, 0.978, and 0.713. For PFS/EFS, median bias-adjusted HRs were 0.52, 0.68, and 1.02, with corresponding probabilities of 0.995, 0.901, and 0.478 (Figures S1 and S2).

### Sensitivity to surgery-timing assumptions

Aggregate timing-bias scenario analysis used the pooled HRs as observed inputs (OS HRobs = 0.33; PFS/EFS HRobs = 0.41). Longer assumed intervals from treatment initiation to surgery and shorter assumed control survival attenuated the pooled estimates toward the null. Across the prespecified grid, the scenario-adjusted estimates remained below 1.0 for both OS and PFS/EFS (Additional file 1: Figure S3 and Table S9).

### Bias profile of the comparative evidence

**Fig. 6:**
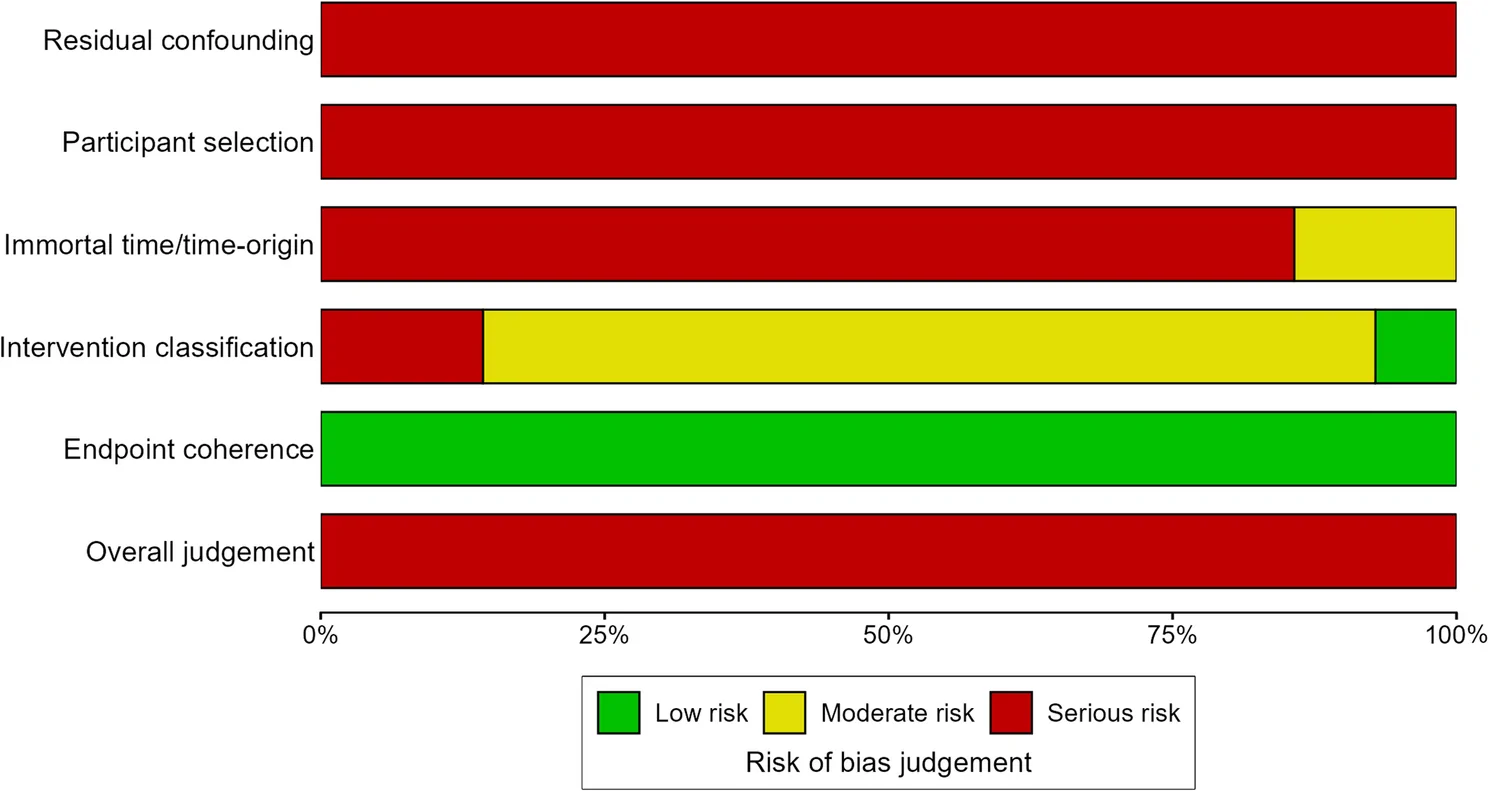
Study-level ROBINS-I-informed risk-of-bias summary. Stacked bars show the proportion of included studies assigned low, moderate, or serious risk across the adapted ROBINS-I-informed domains. Study-level judgments summarize the most serious endpoint-level judgment observed for each study. Endpoint-level judgments are provided in Additional file 1: Tables S4–S5 and Figure S4

ROBINS-I-informed risk-of-bias assessment showed that the comparative evidence remained vulnerable to residual confounding, participant selection, and surgery timing during follow-up. Although the pooled estimates were below 1.0, individual estimates varied in direction and precision, and the ROBINS-I-informed assessment indicated serious residual confounding and timing concerns (Fig. 6; endpoint-level judgments are provided in Additional file 1: Figure S4 and Tables S4–S5).

### Small-study effects and reporting-bias assessment

For OS (k = 14), the contour-enhanced funnel plot showed asymmetry, with several smaller estimates falling in statistically significant protective contours. SE-based Egger regression (t = -3.71, *p* = 0.003) and rank-correlation testing (Kendall’s tau = -0.47, *p* = 0.019) supported funnel-plot asymmetry, whereas the sample-size-based Peters-type regression was not significant (t = 0.77, *p* = 0.456). This pattern suggests possible small-study effects, selective reporting, or true between-study heterogeneity, but it does not prove publication bias. For PFS/EFS (k = 10), the funnel plot and regression diagnostics were treated as descriptive because the synthesis was at the minimum threshold and combined related but non-identical endpoints ( Figure S5 and Table S15).

### Descriptive sensitivity diagnostics

Descriptive supplementary diagnostics described the influence of individual estimates and selected analytic assumptions. These analyses were interpreted descriptively and did not change the overall direction of the pooled estimates (Additional file 1: Tables S10A–S10B). In diagnostics restricted to adjusted or time-dependent estimates, heterogeneity was lower and pooled estimates remained below 1.0 (OS HR 0.21 [95% CI 0.13–0.34]; PFS/EFS HR 0.35 [95% CI 0.24–0.49]).

### Certainty of evidence

The certainty of evidence was rated as very low for both OS and PFS/EFS. For OS, the pooled association favored hepatectomy, but certainty was downgraded. For PFS/EFS, certainty was also rated as very low. Detailed domain-by-domain judgments are provided in Table S12.

## Discussion

In this systematic review and meta-analysis, conversion hepatectomy was associated with improved OS and PFS/EFS compared with non-surgical management in selected patients with initially unresectable HCC. However, substantial between-study heterogeneity and prediction intervals crossing the null indicate that the magnitude of benefit is unlikely to be uniform across clinical settings. These findings support the potential value of hepatectomy after successful conversion therapy, while emphasizing the need to interpret the observed survival advantage in the context of patient selection and time-dependent treatment assignment.

Prior reviews of conversion therapy in HCC have mainly addressed whether initially unresectable tumors can be downstaged to resection, focusing on tumor response, conversion-to-surgery rate, resection rate, safety, and perioperative outcomes [5, 6]. Less attention has been given to the subsequent clinical decision faced after successful downstaging: whether proceeding to hepatectomy adds survival benefit beyond the prognosis already conferred by treatment response, preserved liver function, and favorable tumor biology. The present review addresses this distinction by focusing on comparative survival after conversion or salvage hepatectomy. The pooled associations favored hepatectomy for both OS and PFS/EFS, and E-value analyses suggested that moderate unmeasured confounding alone would be unlikely to fully explain these associations. However, the magnitude of benefit should not be interpreted as the isolated causal effect of surgery. Patients selected for hepatectomy are those who survive to surgical assessment, respond sufficiently to therapy, maintain adequate liver reserve, and remain technically resectable. Accordingly, the observed survival advantage is best interpreted as the outcome of a successful conversion pathway, in which surgical clearance may contribute to the observed survival association within a highly selected responder population. This interpretation is directly relevant to multidisciplinary decision-making: hepatectomy should be considered for carefully selected patients after effective downstaging, while the strength of the survival advantage remains dependent on selection criteria, timing of surgical assessment, and the completeness of bias control in the underlying studies.

The observed survival advantage should be interpreted within the clinical context of post-conversion patient selection. Hepatectomy may improve outcomes by removing residual viable tumor after effective systemic or multimodal therapy, and pathological response after conversion surgery has been associated with recurrence-free and overall survival [36]. However, radiologic response, alpha-fetoprotein decline, radiologic complete response, and pathological complete response represent different dimensions of treatment response and should not be treated as equivalent surrogates for tumor clearance [32, 37]. In clinical practice, patients who proceed to hepatectomy are usually those with sustained disease control, adequate liver-function reserve, preserved performance status, controlled or absent extrahepatic disease, and technically resectable lesions [38]. These factors are themselves prognostic and may partly explain the survival differences between surgical and non-surgical groups. Interpretation is further complicated by the heterogeneity of conversion-intent regimens, including TACE, HAIC, immune checkpoint inhibitors, tyrosine kinase inhibitors, and their combinations. These strategies differ in antitumor activity, depth of response, liver toxicity, and probability of conversion, thereby influencing both eligibility for surgery and subsequent prognosis. Accordingly, the pooled HRs are best understood as reflecting survival within a selected post-conversion surgical pathway rather than a pure surgical treatment effect.

The QBA findings help define the evidential boundaries of this interpretation. These analyses were used not to isolate the causal effect of hepatectomy, but to assess how robust the observed survival associations were to plausible residual bias. Moderate unmeasured confounding was insufficient to fully attenuate the pooled associations, whereas stronger assumptions about surgical candidacy reduced the apparent benefit, particularly for PFS/EFS. Similarly, because hepatectomy was assigned after conversion-intent therapy had begun in several studies, discordance between the start of follow-up and the definition of surgical exposure could introduce immortal-time bias if preoperative event-free time was attributed to the hepatectomy group. The timing-bias scenario analysis should therefore be interpreted as a study-level sensitivity analysis rather than an individual-patient correction. Taken together, the QBA results support the presence of a clinically meaningful survival signal after conversion hepatectomy, but they also indicate that this signal likely reflects the combined effects of effective downstaging, surgical tumor clearance, and selection of patients with favorable biology and preserved clinical reserve, rather than the isolated effect of surgery alone.

Several limitations should be considered. All included studies were non-randomized and varied in unresectability criteria, conversion regimens, endpoint definitions, and adjustment methods. As a result, the pooled HRs should be interpreted as average associations across heterogeneous clinical settings rather than as a uniform treatment effect. Some estimates were unadjusted or reconstructed from Kaplan–Meier curves, and the number and heterogeneity of studies still limited regimen subgroup analyses and made small-study-effect diagnostics exploratory, especially for PFS/EFS. The OS funnel plot suggested asymmetry, which could reflect publication bias, selective reporting, true heterogeneity, or small-study effects; however, the Peters-type sample-size regression was not significant. Although QBA helped assess the potential impact of residual confounding and immortal-time bias, it could not replace individual-level information on surgical eligibility, time to hepatectomy, and reasons for non-resection. These constraints explain the very low certainty of evidence and highlight the need for prospective cohorts, individual-patient-data analyses, registry-based target-trial emulation, or randomized comparisons where equipoise exists.

## Conclusion

Conversion hepatectomy was associated with improved survival in selected patients with initially unresectable HCC after conversion-intent therapy. This association appeared robust to moderate residual confounding, but its magnitude may be influenced by patient selection, timing bias, and small-study effects. Future studies should define surgical eligibility landmarks, time zero, time to hepatectomy, and reasons for non-resection to better distinguish treatment effect from patient selection.

## Supplementary Information

Additional file 1: Supplementary methods, tables and figures. This file contains database-specific search strategies, study characteristics, extracted endpoint-level HRs, ROBINS-I-informed judgments, E-value analyses, QBA assumptions and outputs, sensitivity analyses, small-study-effect and reporting-bias diagnostics (Table S15 and Figure S5), GRADE certainty assessment, a structured comparison with prior systematic reviews (Table S13), and key full-text reports that appeared potentially eligible but were excluded from quantitative synthesis (Table S14).

Additional file 2: PRISMA 2020 checklist: Completed PRISMA 2020 checklist with manuscript locations for each reporting item.

## Data Availability

All data analysed during this study are included in this published article and its additional file. Extracted study-level and endpoint-level data are provided in Additional file 1. Analysis code is available from the corresponding author upon reasonable request.

## Abbreviations

HCC: Hepatocellular carcinoma
HR: Hazard ratio
QBA: Quantitative bias analysis
OS: Overall survival
PFS: Progression-free survival
EFS: Event-free survival
CI: Confidence interval
PD-1: Programmed cell death protein 1
TACE: Transarterial chemoembolization
HAIC: Hepatic arterial infusion chemotherapy
TKI: Tyrosine kinase inhibitor
ICIs: Immune checkpoint inhibitors
ROBINS-I: Risk of Bias in Non-randomized Studies of Interventions
PRISMA: Preferred Reporting Items for Systematic Reviews and Meta-Analyses
PROSPERO: International Prospective Register of Systematic Reviews
KM: Kaplan–Meier
MVA: Multivariable adjustment
TD-Cox: Time-dependent Cox regression
REML: Restricted maximum likelihood
SE: Standard error
RR_EU_: Relative risk of exposure among the unexposed
RR_UY_: Relative risk of the outcome among the unexposed
BF: Bias factor
CR: Complete response
PR: Partial response
AFP: Alpha-fetoprotein
PVTT: Portal vein tumor thrombosis
FOLFOX: Folinic acid, fluorouracil, and oxaliplatin
