# Supplementary material for: Conversion hepatectomy and survival in initially unresectable hepatocellular carcinoma: a systematic review and meta-analysis with quantitative bias analysis
Source: World J Surg Oncol. 2026 Jul 16;24:391. doi: 10.1186/s12957-026-04498-x (PMC13656502; doi:10.1186/s12957-026-04498-x)
Supplement: Supplementary file 1 [file 12957_2026_4498_MOESM2_ESM.docx]

**Additional file 1. Supplementary methods, tables and figures**

**Supplementary methods**

**Methods S1. Study selection and PRISMA flow**

Fourteen comparative survival studies met the eligibility criteria and contributed 24 endpoint-specific HRs comparing hepatectomy with non-hepatectomy management, including 14 OS and 10 PFS/EFS estimates. The updated search and eligibility verification according to the prespecified PICO criteria identified two additional eligible comparative studies, Sun Z 2026 and Wu JY 2024. These studies were incorporated into the included-study ledger, endpoint-level extraction table, main random-effects syntheses, E-value analyses, deterministic and probabilistic QBA, timing-bias scenario analyses, sensitivity analyses, ROBINS-I-informed assessment, and GRADE certainty assessment. All main and supplementary tables and figures have been updated to reflect the 14-study dataset.

**Methods S2. HR extraction and time-origin adjudication**

For each eligible HR, the natural logarithm of the HR was analyzed. The standard error was calculated as SE = [log(U) - log(L)] / 3.92, where U and L denote the upper and lower limits of the reported 95% CI. HR extraction retained endpoint, comparison, model/source type, adjustment status, time origin, exposure timing, source location, and E-value fields. OS and PFS/EFS were analyzed separately.

Time-origin labels were T0 for treatment-initiation anchoring, PC for post-conversion or post-response anchoring, and UNC when the original report did not provide a single unambiguous time origin. HRs derived from Kaplan–Meier curves were retained in the main synthesis and evaluated in supplementary diagnostics.

**Methods S3. Quantitative bias analysis parameters**

Deterministic quantitative bias analysis used the standard maximum bias-factor approach for unmeasured confounding. For each endpoint, the pooled HR from the main random-effects synthesis was used as the observed estimate (HR_obs). RR_EU denoted the maximum relative imbalance in unfavorable surgical candidacy between the non-hepatectomy and hepatectomy groups, and RR_UY denoted the maximum association between unfavorable surgical candidacy and the event. The bias factor was calculated as:

BF = (RR_EU_ × RR_UY_) / (RR_EU_ + RR_UY_ − 1)

For protective associations, the bias-adjusted estimate was calculated as:

HR_adj_ = HR_obs_ × BF

RR_EU_ and RR_UY_ were evaluated from 1.00 to 10.00 in increments of 0.05. Null thresholds identified parameter combinations for which HR_adj reached or exceeded 1.0.

Probabilistic quantitative bias analysis assigned log-normal distributions to RR_EU and RR_UY. Moderate, strong, and extreme surgical-selection confounding scenarios used median RR_EU/RR_UY values of 1.8/2.0, 2.5/3.0, and 4.0/5.0, with sdlog values of 0.25, 0.30, and 0.35, respectively. Unless otherwise specified, RR_EU and RR_UY were sampled independently. Each outcome-scenario combination used 50,000 Monte Carlo iterations. Simulated results were summarized using the median, 95% simulation interval, and the proportion of simulations in which HR_adj remained below 1.0.

Aggregate timing-bias scenario analysis varied the assumed time from treatment initiation to surgery (2, 4, or 6 months) and the assumed median survival of the non-hepatectomy comparator group (6, 12, 18, and 24 months). The comparator event rate was defined as λ_control = log_（2）_ / median survival, with survival time expressed in months. The timing-bias factor was calculated as:

timing factor = exp(λ_control_ × t_surgery_)

where t_surgery_ denotes the assumed time from treatment initiation to surgery in months. The scenario-adjusted HR was calculated as:

HR_timing_ = HR_obs_ × timing factor

This calculation assumes an exponential survival distribution in the comparator group and a fixed time from treatment initiation to surgery. It was used to describe aggregate sensitivity to timing assumptions at the study level.

**Methods S4. Sensitivity analyses and subgroup policy**

No regimen-based subgroup meta-analysis was performed because the number of comparative studies was small and the main heterogeneity concerned estimand structure, time origin, and HR source. Prespecified descriptive diagnostics included leave-one-out analysis, exclusion of Kaplan–Meier-derived estimates, exclusion of extreme HRs, adjusted/time-dependent-only estimates, directly reported HR-only estimates where applicable, and influence diagnostics.

**Methods S5. Small-study-effect and reporting-bias assessment**

**Because the OS synthesis included 14 estimates, we generated a contour-enhanced funnel plot and used SE-based Egger regression, sample-size-based Peters-type regression, and rank-correlation testing to evaluate funnel-plot asymmetry. Because the PFS/EFS synthesis included 10 estimates and combined related but non-identical endpoints, its funnel plot and regression diagnostics were treated as descriptive. Results were used as supplementary diagnostics for GRADE publication-bias judgments and were not used to adjust pooled estimates.**

**Methods S6. Software and reproducibility**

Analyses were performed in R 4.5.3 using prespecified analysis code. Random-effects models, influence diagnostics, and small-study-effect tests were implemented with metafor; primary figures were generated using ggplot2, and Figure S5 was generated using the native metafor funnel function. The analysis script set seed 20260504. Extracted data and code are available from the corresponding author upon reasonable request.

**Table S1. Search strategies, database counts, dates, and verification**

| **Database/platform** | **Records** | **Search/export date** | **Search string / source query** | **Limits/filters** | **Verification** |
| --- | --- | --- | --- | --- | --- |
| PubMed/MEDLINE | 687 | 2026-06-14 | (("Carcinoma, Hepatocellular"[Mesh] OR hepatocellular carcinoma[tiab] OR HCC[tiab] OR liver cancer[tiab]) AND (unresectable[tiab] OR initially unresectable[tiab] OR advanced[tiab]) AND (conversion[tiab] OR downstaging[tiab] OR salvage[tiab] OR neoadjuvant[tiab]) AND (hepatectomy[tiab] OR resection[tiab] OR surgery[tiab])) | No publication-date restriction; human clinical HCC conversion/surgery focus | Search counts and screening decisions were verified during review data extraction. |
| Embase | 1266 | 2026-06-14 | ('hepatocellular carcinoma'/exp OR 'hepatocellular carcinoma':ti,ab OR hcc:ti,ab) AND (unresectable:ti,ab OR 'initially unresectable':ti,ab OR advanced:ti,ab) AND (conversion:ti,ab OR downstaging:ti,ab OR salvage:ti,ab OR neoadjuvant:ti,ab) AND (hepatectomy:ti,ab OR resection:ti,ab OR surgery:ti,ab) | No publication-date restriction; human clinical HCC conversion/surgery focus | Search counts and screening decisions were verified during review data extraction. |
| Web of Science | 2099 | 2026-06-14 | TS=((hepatocellular carcinoma OR HCC OR liver cancer) AND (unresectable OR initially unresectable OR advanced) AND (conversion OR downstaging OR salvage OR neoadjuvant) AND (hepatectomy OR resection OR surgery)) | No publication-date restriction; human clinical HCC conversion/surgery focus | Search counts and screening decisions were verified during review data extraction. |
| Scopus | 406 | 2026-06-14 | TITLE-ABS-KEY((hepatocellular carcinoma OR HCC OR liver cancer) AND (unresectable OR initially unresectable OR advanced) AND (conversion OR downstaging OR salvage OR neoadjuvant) AND (hepatectomy OR resection OR surgery)) | No publication-date restriction; human clinical HCC conversion/surgery focus | Search counts and screening decisions were verified during review data extraction. |
| CENTRAL | 392 | 2026-06-14 | (hepatocellular carcinoma OR HCC OR liver cancer) AND (unresectable OR initially unresectable OR advanced) AND (conversion OR downstaging OR salvage OR neoadjuvant) AND (hepatectomy OR resection OR surgery) | No publication-date restriction; human clinical HCC conversion/surgery focus | Search counts and screening decisions were verified during review data extraction. |

Note: Database counts and search concepts are reported in Table S1.

**Table S2. Characteristics of the 14 included comparative survival studies**

| **No.** | **Study** | **Design** | **Region** | **Starting cohort N** | **Surgery N** | **Non-surgery N** | **Endpoints** | **Comparison** | **DOI** |
| --- | --- | --- | --- | --- | --- | --- | --- | --- | --- |
| 1 | Chi QY 2025 | Real-world | China | 74 | 29 | 45 | OS; PFS/EFS | surgical resection vs no surgical resection | 10.1186/s12885-025-14543-9 |
| 2 | Gao W 2025 | Phase II | China | 36 | 18 | 18 | OS; PFS/EFS | surgery vs non-surgical group | 10.1016/j.eclinm.2025.103217 |
| 3 | Goto Y 2025 | Non-randomized comparative cohort | Japan | 153 | 24 | 129 | OS; PFS/EFS | conversion cases vs unresectable cases after HAIC | 10.1002/ags3.70034 |
| 4 | Lin S 2025 | Non-randomized comparative cohort | China | 301 | 63 | 238 | OS; PFS/EFS | successful surgery vs no successful surgery | 10.1159/000547725 |
| 5 | Piao M 2025 | Retrospective | China | 613 | 74 | 539 | OS; PFS/EFS | conversion surgery vs continued nonsurgical treatment after conversion criteria | 10.1159/000543994 |
| 6 | Xue Z 2025 | Real-world | China | 79 | 20 | 59 | OS | hepatectomy vs no hepatectomy | 10.1038/s41598-025-88813-8 |
| 7 | Yang DL 2025 | Multicenter retrospective cohort | China | 1262 | 356 | 906 | OS | subsequent hepatectomy vs other downstream treatments | 10.1016/j.jhepr.2025.101587 |
| 8 | Lu YC 2024 | Real-world | China | 51 | 13 | 38 | OS; PFS/EFS | surgery vs non-surgery group | 10.3389/fimmu.2024.1471017 |
| 9 | Pang B 2024 | Real-world | China | 62 | 17 | 45 | OS; PFS/EFS | successful conversion to surgical resection vs unsuccessful conversion | 10.1016/j.intimp.2024.112492 |
| 10 | Wang L 2024 | Retrospective multicenter cohort | China | 93 | 44 | 49 | OS; PFS/EFS | salvage surgery vs non-surgery after successful conversion | 10.1093/oncolo/oyae032 |
| 11 | Li X 2023 | Phase II | China | 94 | 32 | 62 | OS; PFS/EFS | successful conversion surgery vs no successful conversion surgery | 10.3389/fonc.2023.1110689 |
| 12 | Zhu XD 2023 | Non-randomized comparative cohort | China | 101 | 24 | 77 | OS | hepatectomy vs no hepatectomy after systemic therapy | 10.1245/s10434-022-12530-z |
| 13 | Sun Z 2026 | Retrospective | China | 67 | 30 | 37 | OS; PFS | surgical resection vs non-surgical treatment after ICI+TKI conversion | 10.1186/s12876-026-04677-w |
| 14 | Wu JY 2024 | Multicenter retrospective | China | 144 | 91 | 53 | OS; PFS reported, but only OS provided an extractable HR for quantitative synthesis | salvage surgery vs non-salvage surgery after TACE+lenvatinib+anti-PD-1 | 10.1245/s10434-024-14944-3 |

Note: Order follows the study extraction table.

Note: Table S2 was updated to include 14 studies (Sun Z 2026 and Wu JY 2024 added through updated eligibility verification according to the prespecified PICO criteria). Downstream supplementary tables (S3 and beyond) have been updated to the 14-study quantitative synthesis.

**Table S3. Extracted comparative HRs for hepatectomy versus non-hepatectomy management**

| **Study** | **Endpoint** | **HR (95% CI)** | **Comparison** | **Model/HR source** | **Adjustment** | **Time origin** | **Exposure timing** | **Source location** |
| --- | --- | --- | --- | --- | --- | --- | --- | --- |
| Chi QY 2025 | OS | 0.19 (0.06–0.66) | surgical resection vs no surgical resection | MVA Cox | Adjusted | T0 | Post-baseline surgical resection status | Page 9; Table 4 |
| Chi QY 2025 | PFS | 0.34 (0.12–0.97) | surgical resection vs no surgical resection | MVA Cox | Adjusted | UNC | Post-baseline surgical resection status | Page 9; Table S8 |
| Gao W 2025 | OS | 0.24 (0.06–0.97) | surgery vs non-surgical group | MVA Cox | Adjusted | T0 | Post-baseline surgery status | Page 5; Supplemental Tables S8/S9 referenced |
| Gao W 2025 | EFS | 0.10 (0.02–0.51) | surgery vs non-surgical group | MVA Cox | Adjusted | T0 | Post-baseline surgery status | Page 5; Supplemental Tables S8/S9 referenced |
| Goto Y 2025 | OS | 0.44 (0.27–0.72) | conversion cases vs unresectable cases after HAIC | KM-derived | Unadjusted | UNC | Post-baseline conversion surgery status | Page 8; Fig. 4a |
| Goto Y 2025 | PFS | 0.42 (0.25–0.70) | conversion cases vs unresectable cases after HAIC | KM-derived | Unadjusted | UNC | Post-baseline conversion surgery status | Page 8; Fig. 4b |
| Lin S 2025 | OS | 0.17 (0.07–0.40) | successful surgery vs no successful surgery | TD-Cox | Adjusted | UNC | Time-dependent surgery variable reported | Page 17; Fig. 7a; Table 5 |
| Lin S 2025 | PFS | 0.37 (0.22–0.61) | successful surgery vs no successful surgery | TD-Cox | Adjusted | UNC | Time-dependent surgery variable reported | Page 17; Fig. 7b; Table 5 |
| Piao M 2025 | OS | 0.26 (0.14–0.46) | conversion surgery vs continued nonsurgical treatment after conversion criteria | KM-derived | Unadjusted | T0 | Landmark-like post-conversion criteria group, surgery chosen after conversion criteria | Page 12; Figs. 4-5 |
| Piao M 2025 | PFS | 0.39 (0.24–0.63) | conversion surgery vs continued nonsurgical treatment after conversion criteria | KM-derived | Unadjusted | T0 | Landmark-like post-conversion criteria group, surgery chosen after conversion criteria | Pages 11-12; Figs. 4-5 |
| Xue Z 2025 | OS | 0.39 (0.18–0.85) | hepatectomy vs no hepatectomy | MVA Cox | Adjusted | T0 | Post-baseline hepatectomy status | Page 1; abstract |
| Yang DL 2025 | OS | 0.36 (0.18–0.74) | subsequent hepatectomy vs other downstream treatments | Unadjusted Cox; rCR-anchored | Unadjusted | rCR | Subsequent hepatectomy after rCR | Page 8; Fig. S10 referenced |
| Lu YC 2024 | OS | 0.12 (0.02–0.63) | surgery vs non-surgery group | KM-derived | Unadjusted | UNC | Post-baseline surgery status | Page 4; Fig. 3C |
| Lu YC 2024 | PFS | 0.20 (0.07–0.58) | surgery vs non-surgery group | KM-derived | Unadjusted | UNC | Post-baseline surgery status | Page 4; Fig. 3D |
| Pang B 2024 | OS | 0.52 (0.28–0.99) | successful conversion to surgical resection vs unsuccessful conversion | KM-derived | Unadjusted | UNC | Post-baseline successful conversion/surgery status | Page 6; Fig. 3D |
| Pang B 2024 | PFS | 0.44 (0.24–0.81) | successful conversion to surgical resection vs unsuccessful conversion | KM-derived | Unadjusted | UNC | Post-baseline successful conversion/surgery status | Page 6; Fig. 3C |
| Wang L 2024 | OS | 1.24 (0.78–1.97) | salvage surgery vs non-surgery after successful conversion | KM-derived | Unadjusted | T0 | Post-baseline salvage surgery status | Page 5; Figure 1A |
| Wang L 2024 | PFS | 1.25 (0.79–1.98) | salvage surgery vs non-surgery after successful conversion | KM-derived | Unadjusted | PC | Post-baseline salvage surgery status | Page 5; Figure 1B |
| Li X 2023 | OS | 0.15 (0.04–0.55) | successful conversion surgery vs no successful conversion surgery | MVA Cox | Adjusted | T0 | Post-baseline successful conversion surgery status | Page 6; Table 5 |
| Li X 2023 | PFS | 0.25 (0.10–0.62) | successful conversion surgery vs no successful conversion surgery | MVA Cox | Adjusted | T0 | Post-baseline successful conversion surgery status | Page 6; Table 4 |
| Zhu XD 2023 | OS | 0.05 (0.01–0.36) | hepatectomy vs no hepatectomy after systemic therapy | MVA Cox | Adjusted | T0 | Post-baseline hepatectomy status | Page 1; abstract; Page 3 methods |
| Sun Z 2026 | OS | 0.56 (0.25–1.43) | surgical resection vs non-surgical treatment after ICI+TKI conversion | Univariate Cox forest plot | Unadjusted | PC | Post-baseline surgical resection status | Page 5; Fig. 3A |
| Sun Z 2026 | PFS | 0.46 (0.23–0.91) | surgical resection vs non-surgical treatment after ICI+TKI conversion | Multivariable Cox | Adjusted | PC | Post-baseline surgical resection status | Page 5; Results paragraph |
| Wu JY 2024 | OS | 0.25 (0.09–0.68) | salvage surgery vs non-salvage surgery after TACE+lenvatinib+anti-PD-1 | KM-derived approximate | Unadjusted; approximate | T0 | Post-baseline salvage surgery status | Page 1; abstract; Page 6; Fig. 2A |

Note: PC denotes post-conversion/post-response anchoring; for Sun Z 2026, PC corresponded to the successful-conversion date. T0 denotes initiation of conversion therapy; for Wu JY 2024, follow-up was anchored at treatment initiation.

Note: Covariate-level adjustment sets were not itemized in the extraction table; this table preserves adjustment status and model/source type from the extraction table.

**Table S4. ROBINS-I-informed domain-level risk-of-bias judgments**

| **Study** | **Endpoint** | **Confounding** | **Participant selection** | **Immortal time** | **Intervention classification** | **Endpoint coherence** | **Overall** |
| --- | --- | --- | --- | --- | --- | --- | --- |
| Chi QY 2025 | OS | Serious | Serious | Serious | Moderate | Low | Serious |
| Chi QY 2025 | PFS/EFS | Serious | Serious | Serious | Moderate | Low | Serious |
| Gao W 2025 | OS | Serious | Serious | Serious | Moderate | Low | Serious |
| Gao W 2025 | PFS/EFS | Serious | Serious | Serious | Moderate | Low | Serious |
| Goto Y 2025 | OS | Serious | Serious | Serious | Serious | Low | Serious |
| Goto Y 2025 | PFS/EFS | Serious | Serious | Serious | Serious | Low | Serious |
| Lin S 2025 | OS | Serious | Serious | Moderate | Low | Low | Serious |
| Lin S 2025 | PFS/EFS | Serious | Serious | Moderate | Low | Low | Serious |
| Piao M 2025 | OS | Serious | Serious | Serious | Moderate | Low | Serious |
| Piao M 2025 | PFS/EFS | Serious | Serious | Serious | Moderate | Low | Serious |
| Xue Z 2025 | OS | Serious | Serious | Serious | Moderate | Low | Serious |
| Yang DL 2025 | OS | Serious | Serious | Serious | Moderate | Low | Serious |
| Lu YC 2024 | OS | Serious | Serious | Serious | Moderate | Low | Serious |
| Lu YC 2024 | PFS/EFS | Serious | Serious | Serious | Moderate | Low | Serious |
| Pang B 2024 | OS | Serious | Serious | Serious | Serious | Low | Serious |
| Pang B 2024 | PFS/EFS | Serious | Serious | Serious | Serious | Low | Serious |
| Wang L 2024 | OS | Serious | Serious | Serious | Moderate | Low | Serious |
| Wang L 2024 | PFS/EFS | Serious | Serious | Serious | Moderate | Low | Serious |
| Li X 2023 | OS | Serious | Serious | Serious | Moderate | Low | Serious |
| Li X 2023 | PFS/EFS | Serious | Serious | Serious | Moderate | Low | Serious |
| Zhu XD 2023 | OS | Serious | Serious | Serious | Moderate | Low | Serious |
| Sun Z 2026 | OS | Serious | Serious | Serious | Moderate | Low | Serious |
| Sun Z 2026 | PFS/EFS | Serious | Serious | Serious | Moderate | Low | Serious |
| Wu JY 2024 | OS | Serious | Serious | Moderate | Moderate | Low | Serious |

Note: Judgements emphasize the dominant non-randomized surgery-selection and timing domains.

**Table S5. ROBINS-I support-for-judgement notes**

| **Study** | **Endpoint** | **Overall judgement** | **Support-for-judgement note** |
| --- | --- | --- | --- |
| Chi QY 2025 | OS | Serious | surgical resection vs no surgical resection; exposure timing: Post-baseline surgical resection status; time origin: T0; HR source: MVA Cox at Page 9; Table 4. Overall judgement driven by non-randomized surgical-candidacy selection and post-baseline treatment classification. |
| Chi QY 2025 | PFS/EFS | Serious | surgical resection vs no surgical resection; exposure timing: Post-baseline surgical resection status; time origin: UNC; HR source: MVA Cox at Page 9; Table S8. Overall judgement driven by non-randomized surgical-candidacy selection and post-baseline treatment classification. |
| Gao W 2025 | OS | Serious | surgery vs non-surgical group; exposure timing: Post-baseline surgery status; time origin: T0; HR source: MVA Cox at Page 5; Supplemental Tables S8/S9 referenced. Overall judgement driven by non-randomized surgical-candidacy selection and post-baseline treatment classification. |
| Gao W 2025 | PFS/EFS | Serious | surgery vs non-surgical group; exposure timing: Post-baseline surgery status; time origin: T0; HR source: MVA Cox at Page 5; Supplemental Tables S8/S9 referenced. Overall judgement driven by non-randomized surgical-candidacy selection and post-baseline treatment classification. |
| Goto Y 2025 | OS | Serious | conversion cases vs unresectable cases after HAIC; exposure timing: Post-baseline conversion surgery status; time origin: UNC; HR source: KM-derived at Page 8; Fig. 4a. Overall judgement driven by non-randomized surgical-candidacy selection and post-baseline treatment classification. |
| Goto Y 2025 | PFS/EFS | Serious | conversion cases vs unresectable cases after HAIC; exposure timing: Post-baseline conversion surgery status; time origin: UNC; HR source: KM-derived at Page 8; Fig. 4b. Overall judgement driven by non-randomized surgical-candidacy selection and post-baseline treatment classification. |
| Lin S 2025 | OS | Serious | successful surgery vs no successful surgery; exposure timing: Time-dependent surgery variable reported; time origin: UNC; HR source: TD-Cox at Page 17; Fig. 7a; Table 5. Overall judgement driven by non-randomized surgical-candidacy selection and post-baseline treatment classification. |
| Lin S 2025 | PFS/EFS | Serious | successful surgery vs no successful surgery; exposure timing: Time-dependent surgery variable reported; time origin: UNC; HR source: TD-Cox at Page 17; Fig. 7b; Table 5. Overall judgement driven by non-randomized surgical-candidacy selection and post-baseline treatment classification. |
| Piao M 2025 | OS | Serious | conversion surgery vs continued nonsurgical treatment after conversion criteria; exposure timing: Landmark-like post-conversion criteria group, surgery chosen after conversion criteria; time origin: T0; HR source: KM-derived at Page 12; Figs. 4-5. Overall judgement driven by non-randomized surgical-candidacy selection and post-baseline treatment classification. |
| Piao M 2025 | PFS/EFS | Serious | conversion surgery vs continued nonsurgical treatment after conversion criteria; exposure timing: Landmark-like post-conversion criteria group, surgery chosen after conversion criteria; time origin: T0; HR source: KM-derived at Pages 11-12; Figs. 4-5. Overall judgement driven by non-randomized surgical-candidacy selection and post-baseline treatment classification. |
| Xue Z 2025 | OS | Serious | hepatectomy vs no hepatectomy; exposure timing: Post-baseline hepatectomy status; time origin: T0; HR source: MVA Cox at Page 1; abstract. Overall judgement driven by non-randomized surgical-candidacy selection and post-baseline treatment classification. |
| Yang DL 2025 | OS | Serious | subsequent hepatectomy vs other downstream treatments; exposure timing: subsequent hepatectomy after rCR; time origin: rCR; HR source: Unadjusted Cox; rCR-anchored. Overall judgement driven by serious non-randomized surgical-candidacy selection and post-baseline treatment classification. |
| Lu YC 2024 | OS | Serious | surgery vs non-surgery group; exposure timing: Post-baseline surgery status; time origin: UNC; HR source: KM-derived at Page 4; Fig. 3C. Overall judgement driven by non-randomized surgical-candidacy selection and post-baseline treatment classification. |
| Lu YC 2024 | PFS/EFS | Serious | surgery vs non-surgery group; exposure timing: Post-baseline surgery status; time origin: UNC; HR source: KM-derived at Page 4; Fig. 3D. Overall judgement driven by non-randomized surgical-candidacy selection and post-baseline treatment classification. |
| Pang B 2024 | OS | Serious | successful conversion to surgical resection vs unsuccessful conversion; exposure timing: Post-baseline successful conversion/surgery status; time origin: UNC; HR source: KM-derived at Page 6; Fig. 3D. Overall judgement driven by non-randomized surgical-candidacy selection and post-baseline treatment classification. |
| Pang B 2024 | PFS/EFS | Serious | successful conversion to surgical resection vs unsuccessful conversion; exposure timing: Post-baseline successful conversion/surgery status; time origin: UNC; HR source: KM-derived at Page 6; Fig. 3C. Overall judgement driven by non-randomized surgical-candidacy selection and post-baseline treatment classification. |
| Wang L 2024 | OS | Serious | salvage surgery vs non-surgery after successful conversion; exposure timing: Post-baseline salvage surgery status; time origin: T0; HR source: KM-derived at Page 5; Figure 1A. Overall judgement driven by non-randomized surgical-candidacy selection and post-baseline treatment classification. |
| Wang L 2024 | PFS/EFS | Serious | salvage surgery vs non-surgery after successful conversion; exposure timing: Post-baseline salvage surgery status; time origin: PC; HR source: KM-derived at Page 5; Figure 1B. Overall judgement driven by non-randomized surgical-candidacy selection and post-baseline treatment classification. |
| Li X 2023 | OS | Serious | successful conversion surgery vs no successful conversion surgery; exposure timing: Post-baseline successful conversion surgery status; time origin: T0; HR source: MVA Cox at Page 6; Table 5. Overall judgement driven by non-randomized surgical-candidacy selection and post-baseline treatment classification. |
| Li X 2023 | PFS/EFS | Serious | successful conversion surgery vs no successful conversion surgery; exposure timing: Post-baseline successful conversion surgery status; time origin: T0; HR source: MVA Cox at Page 6; Table 4. Overall judgement driven by non-randomized surgical-candidacy selection and post-baseline treatment classification. |
| Zhu XD 2023 | OS | Serious | hepatectomy vs no hepatectomy after systemic therapy; exposure timing: Post-baseline hepatectomy status; time origin: T0; HR source: MVA Cox at Page 1; abstract; Page 3 methods. Overall judgement driven by non-randomized surgical-candidacy selection and post-baseline treatment classification. |
| Sun Z 2026 | OS | Serious | surgical resection vs non-surgical treatment after ICI+TKI conversion; exposure timing: Post-baseline surgical resection status; time origin: From successful conversion date; HR source: Univariate Cox forest plot at Page 5; Fig. 3A. Overall judgement driven by non-randomized surgical-candidacy selection and post-baseline treatment classification. |
| Sun Z 2026 | PFS/EFS | Serious | surgical resection vs non-surgical treatment after ICI+TKI conversion; exposure timing: Post-baseline surgical resection status; time origin: From successful conversion date; HR source: Multivariable Cox at Page 5; Results paragraph. Overall judgement driven by non-randomized surgical-candidacy selection and post-baseline treatment classification. |
| Wu JY 2024 | OS | Serious | salvage surgery vs non-salvage surgery after TACE+lenvatinib+anti-PD-1; exposure timing: Post-baseline salvage surgery status; time origin: From initiation of conversion therapy; HR source: KM-derived approximate from published survival rates. Overall judgement driven by non-randomized surgical-candidacy selection and approximate HR derivation. |

**Table S6. E-values for study-specific and pooled estimates**

| Study/estimate | Endpoint | HR (95% CI) | E-value | CI-limit E-value |
| --- | --- | --- | --- | --- |
| Chi QY 2025 | OS | 0.19 (0.06–0.66) | 9.78 | 2.41 |
| Chi QY 2025 | PFS/EFS | 0.34 (0.12–0.97) | 5.40 | 1.22 |
| Gao W 2025 | OS | 0.24 (0.06–0.97) | 7.80 | 1.21 |
| Gao W 2025 | PFS/EFS | 0.10 (0.02–0.51) | 19.49 | 3.33 |
| Goto Y 2025 | OS | 0.44 (0.27–0.72) | 3.93 | 2.12 |
| Goto Y 2025 | PFS/EFS | 0.42 (0.25–0.70) | 4.19 | 2.19 |
| Lin S 2025 | OS | 0.17 (0.07–0.40) | 11.24 | 4.44 |
| Lin S 2025 | PFS/EFS | 0.37 (0.22–0.61) | 4.85 | 2.66 |
| Piao M 2025 | OS | 0.26 (0.14–0.46) | 7.15 | 3.77 |
| Piao M 2025 | PFS/EFS | 0.39 (0.24–0.63) | 4.57 | 2.55 |
| Xue Z 2025 | OS | 0.39 (0.18–0.85) | 4.59 | 1.64 |
| Yang DL 2025 | OS | 0.36 (0.18–0.74) | 5.00 | 2.04 |
| Lu YC 2024 | OS | 0.12 (0.02–0.63) | 16.15 | 2.57 |
| Lu YC 2024 | PFS/EFS | 0.20 (0.07–0.58) | 9.47 | 2.85 |
| Pang B 2024 | OS | 0.52 (0.28–0.99) | 3.23 | 1.12 |
| Pang B 2024 | PFS/EFS | 0.44 (0.24–0.81) | 3.93 | 1.78 |
| Wang L 2024 | OS | 1.24 (0.78–1.97) | 1.78 | 1.00 |
| Wang L 2024 | PFS/EFS | 1.25 (0.79–1.98) | 1.82 | 1.00 |
| Li X 2023 | OS | 0.15 (0.04–0.55) | 13.09 | 3.01 |
| Li X 2023 | PFS/EFS | 0.25 (0.10–0.62) | 7.53 | 2.60 |
| Zhu XD 2023 | OS | 0.05 (0.01–0.36) | 39.49 | 4.92 |
| Sun Z 2026 | OS | 0.56 (0.25–1.43) | 3.00 | 1.00 |
| Sun Z 2026 | PFS/EFS | 0.46 (0.23–0.91) | 3.82 | 1.43 |
| Wu JY 2024 | OS | 0.25 (0.09–0.68) | 7.46 | 2.30 |
| Pooled random-effects estimate | Overall survival | 0.33 (0.22–0.48) | 5.54 | 3.58 |
| Pooled random-effects estimate | Progression/event-free survival | 0.41 (0.28–0.59) | 4.34 | 2.81 |

*Note: For protective associations, inverse HRs were used before applying the standard E-value formula; the confidence-limit E-value used the confidence bound closest to the null.*

**Table S7. Deterministic tipping-point quantitative bias analysis grid summary**

| **Outcome** | **RR_EU fixed** | **Minimum RR_UY reaching HR_adj >= 1** | **Grid definition** |
| --- | --- | --- | --- |
| OS | 1.5 | Not reached within 1–10 | RR_EU and RR_UY 1.00–10.00 by 0.05; BF=(RR_EU*RR_UY)/(RR_EU+RR_UY-1) |
| OS | 2 | Not reached within 1–10 | RR_EU and RR_UY 1.00–10.00 by 0.05; BF=(RR_EU*RR_UY)/(RR_EU+RR_UY-1) |
| OS | 2.5 | Not reached within 1–10 | RR_EU and RR_UY 1.00–10.00 by 0.05; BF=(RR_EU*RR_UY)/(RR_EU+RR_UY-1) |
| OS | 3 | Not reached within 1–10 | RR_EU and RR_UY 1.00–10.00 by 0.05; BF=(RR_EU*RR_UY)/(RR_EU+RR_UY-1) |
| OS | 4 | 9.53 | RR_EU and RR_UY 1.00–10.00 by 0.05; BF=(RR_EU*RR_UY)/(RR_EU+RR_UY-1) |
| OS | 5 | 6.22 | RR_EU and RR_UY 1.00–10.00 by 0.05; BF=(RR_EU*RR_UY)/(RR_EU+RR_UY-1) |
| OS | 6 | 5.14 | RR_EU and RR_UY 1.00–10.00 by 0.05; BF=(RR_EU*RR_UY)/(RR_EU+RR_UY-1) |
| OS | 8 | 4.30 | RR_EU and RR_UY 1.00–10.00 by 0.05; BF=(RR_EU*RR_UY)/(RR_EU+RR_UY-1) |
| OS | 10 | 3.94 | RR_EU and RR_UY 1.00–10.00 by 0.05; BF=(RR_EU*RR_UY)/(RR_EU+RR_UY-1) |
| PFS/EFS | 1.5 | Not reached within 1–10 | RR_EU and RR_UY 1.00–10.00 by 0.05; BF=(RR_EU*RR_UY)/(RR_EU+RR_UY-1) |
| PFS/EFS | 2 | Not reached within 1–10 | RR_EU and RR_UY 1.00–10.00 by 0.05; BF=(RR_EU*RR_UY)/(RR_EU+RR_UY-1) |
| PFS/EFS | 2.5 | Not reached within 1–10 | RR_EU and RR_UY 1.00–10.00 by 0.05; BF=(RR_EU*RR_UY)/(RR_EU+RR_UY-1) |
| PFS/EFS | 3 | 8.95 | RR_EU and RR_UY 1.00–10.00 by 0.05; BF=(RR_EU*RR_UY)/(RR_EU+RR_UY-1) |
| PFS/EFS | 4 | 4.75 | RR_EU and RR_UY 1.00–10.00 by 0.05; BF=(RR_EU*RR_UY)/(RR_EU+RR_UY-1) |
| PFS/EFS | 5 | 3.85 | RR_EU and RR_UY 1.00–10.00 by 0.05; BF=(RR_EU*RR_UY)/(RR_EU+RR_UY-1) |
| PFS/EFS | 6 | 3.46 | RR_EU and RR_UY 1.00–10.00 by 0.05; BF=(RR_EU*RR_UY)/(RR_EU+RR_UY-1) |
| PFS/EFS | 8 | 3.09 | RR_EU and RR_UY 1.00–10.00 by 0.05; BF=(RR_EU*RR_UY)/(RR_EU+RR_UY-1) |
| PFS/EFS | 10 | 2.92 | RR_EU and RR_UY 1.00–10.00 by 0.05; BF=(RR_EU*RR_UY)/(RR_EU+RR_UY-1) |

Note: The full deterministic grid contains all RR_EU x RR_UY combinations from 1.00 to 10.00 by 0.05 and is available from the corresponding author upon reasonable request.

**Table S8. Probabilistic QBA assumptions, distributions, and outputs**

| **Outcome** | **Scenario** | **RR_EU median** | **RR_UY median** | **sdlog** | **Iterations** | **Median adjusted HR** | **95% simulation interval** | **Pr(HR_adj < 1)** |
| --- | --- | --- | --- | --- | --- | --- | --- | --- |
| Overall survival | Moderate surgical-selection confounding | 1.8 | 2.0 | 0.25 | 50000 | 0.42 | 0.26–0.69 | 1.000 |
| Progression/event-free survival | Moderate surgical-selection confounding | 1.8 | 2.0 | 0.25 | 50000 | 0.52 | 0.32–0.86 | 0.995 |
| Overall survival | Strong surgical-selection confounding | 2.5 | 3.0 | 0.3 | 50000 | 0.55 | 0.30–0.99 | 0.978 |
| Progression/event-free survival | Strong surgical-selection confounding | 2.5 | 3.0 | 0.3 | 50000 | 0.68 | 0.38–1.22 | 0.901 |
| Overall survival | Extreme surgical-selection confounding | 4.0 | 5.0 | 0.35 | 50000 | 0.82 | 0.41–1.63 | 0.713 |
| Progression/event-free survival | Extreme surgical-selection confounding | 4.0 | 5.0 | 0.35 | 50000 | 1.02 | 0.51–2.02 | 0.478 |

*Note: Seed=20260504; RR_EU and RR_UY sampled from log-normal distributions using the listed medians and sdlog values.*

**Table S9. Aggregate timing-bias scenario analysis**

| **Outcome** | **Months to surgery** | **Comparator median survival (mo)** | **Pooled HR** | **Timing bias factor** | **Timing-adjusted HR** |
| --- | --- | --- | --- | --- | --- |
| OS | 2 | 6 | 0.329 | 1.26 | 0.410 |
| OS | 2 | 12 | 0.329 | 1.12 | 0.370 |
| OS | 2 | 18 | 0.329 | 1.08 | 0.350 |
| OS | 2 | 24 | 0.329 | 1.06 | 0.350 |
| OS | 4 | 6 | 0.329 | 1.59 | 0.520 |
| OS | 4 | 12 | 0.329 | 1.26 | 0.410 |
| OS | 4 | 18 | 0.329 | 1.17 | 0.380 |
| OS | 4 | 24 | 0.329 | 1.12 | 0.370 |
| OS | 6 | 6 | 0.329 | 2.00 | 0.660 |
| OS | 6 | 12 | 0.329 | 1.41 | 0.460 |
| OS | 6 | 18 | 0.329 | 1.26 | 0.410 |
| OS | 6 | 24 | 0.329 | 1.19 | 0.390 |
| PFS/EFS | 2 | 6 | 0.408 | 1.26 | 0.510 |
| PFS/EFS | 2 | 12 | 0.408 | 1.12 | 0.460 |
| PFS/EFS | 2 | 18 | 0.408 | 1.08 | 0.440 |
| PFS/EFS | 2 | 24 | 0.408 | 1.06 | 0.430 |
| PFS/EFS | 4 | 6 | 0.408 | 1.59 | 0.650 |
| PFS/EFS | 4 | 12 | 0.408 | 1.26 | 0.510 |
| PFS/EFS | 4 | 18 | 0.408 | 1.17 | 0.480 |
| PFS/EFS | 4 | 24 | 0.408 | 1.12 | 0.460 |
| PFS/EFS | 6 | 6 | 0.408 | 2.00 | 0.820 |
| PFS/EFS | 6 | 12 | 0.408 | 1.41 | 0.580 |
| PFS/EFS | 6 | 18 | 0.408 | 1.26 | 0.510 |
| PFS/EFS | 6 | 24 | 0.408 | 1.19 | 0.490 |

*Note: This represents an aggregate study-level sensitivity analysis.*

**Table S10A. Supplementary descriptive diagnostics: leave-one-out and influence analyses**

| **Outcome** | **Study omitted** | **Pooled HR after omission** | **Cook's distance** | **Weight (%)** |
| --- | --- | --- | --- | --- |
| OS | Chi QY 2025 | 0.34 (0.23–0.50) | 0.020 | 5.5 |
| OS | Gao W 2025 | 0.33 (0.22–0.49) | 0.000 | 4.6 |
| OS | Goto Y 2025 | 0.31 (0.21–0.48) | 0.060 | 10.2 |
| OS | Lin S 2025 | 0.35 (0.24–0.51) | 0.090 | 7.5 |
| OS | Piao M 2025 | 0.33 (0.22–0.50) | 0.000 | 9.4 |
| OS | Xue Z 2025 | 0.32 (0.21–0.48) | 0.020 | 8.1 |
| OS | Yang DL 2025 | 0.32 (0.21–0.49) | 0.010 | 8.6 |
| OS | Lu YC 2024 | 0.34 (0.23–0.50) | 0.050 | 3.7 |
| OS | Pang B 2024 | 0.31 (0.21–0.47) | 0.090 | 9.2 |
| OS | Wang L 2024 | 0.31 (0.24–0.41) | 0.060 | 10.4 |
| OS | Li X 2023 | 0.34 (0.23–0.51) | 0.050 | 5.0 |
| OS | Zhu XD 2023 | 0.35 (0.25–0.51) | 0.160 | 3.3 |
| OS | Sun Z 2026 | 0.31 (0.21–0.47) | 0.080 | 7.9 |
| OS | Wu JY 2024 | 0.33 (0.22–0.50) | 0.000 | 6.6 |
| PFS/EFS | Chi QY 2025 | 0.41 (0.28–0.60) | 0.000 | 7.0 |
| PFS/EFS | Gao W 2025 | 0.43 (0.30–0.62) | 0.110 | 3.8 |
| PFS/EFS | Goto Y 2025 | 0.40 (0.26–0.60) | 0.010 | 12.7 |
| PFS/EFS | Lin S 2025 | 0.41 (0.27–0.62) | 0.000 | 12.8 |
| PFS/EFS | Piao M 2025 | 0.40 (0.27–0.61) | 0.000 | 13.1 |
| PFS/EFS | Lu YC 2024 | 0.43 (0.30–0.62) | 0.090 | 6.9 |
| PFS/EFS | Pang B 2024 | 0.40 (0.26–0.60) | 0.020 | 11.5 |
| PFS/EFS | Wang L 2024 | 0.37 (0.30–0.46) | 0.280 | 13.4 |
| PFS/EFS | Li X 2023 | 0.43 (0.29–0.62) | 0.050 | 8.2 |
| PFS/EFS | Sun Z 2026 | 0.40 (0.26–0.60) | 0.020 | 10.5 |

**Table S10B. Supplementary descriptive diagnostics: restricted-set summaries**

| **Outcome** | **Scenario** | **HR rows** | **Pooled HR (95% CI)** | **I² (%)** | **τ²** |
| --- | --- | --- | --- | --- | --- |
| OS | All hepatectomy-comparison HRs | 14 | 0.33 (0.22–0.48) | 65.7 | 0.307 |
| OS | Adjusted or TD-Cox only | 6 | 0.21 (0.13–0.34) | 14.0 | 0.053 |
| OS | Excluding KM-derived estimates | 8 | 0.27 (0.18–0.40) | 21.9 | 0.072 |
| OS | Excluding alternative/unclear time origin | 9 | 0.32 (0.18–0.56) | 71.4 | 0.479 |
| OS | Excluding extreme HR <0.15 | 12 | 0.37 (0.26–0.54) | 64.0 | 0.250 |
| OS | Wang L 2024 influence check | 13 | 0.31 (0.24–0.41) | 18.4 | 0.042 |
| PFS/EFS | All hepatectomy-comparison HRs | 10 | 0.41 (0.28–0.59) | 64.3 | 0.197 |
| PFS/EFS | Adjusted or TD-Cox only | 5 | 0.35 (0.24–0.49) | 0.0 | 0.000 |
| PFS/EFS | Excluding KM-derived estimates | 5 | 0.35 (0.24–0.49) | 0.0 | 0.000 |
| PFS/EFS | Excluding alternative/unclear time origin | 5 | 0.42 (0.21–0.87) | 81.4 | 0.502 |

Note: Restricted-set definitions were applied at the endpoint-HR level. The 'adjusted or time-dependent Cox only' set included endpoint-specific HRs with multivariable adjustment or time-dependent Cox modeling. The 'excluding KM-derived estimates' set excluded HRs reconstructed from Kaplan–Meier curves, survival rates, or other indirect information. The 'excluding alternative/unclear time origin' set excluded estimates with unclear (UNC) or radiologic-complete-response (rCR) time origins. OS adjusted/TD-Cox: Chi QY 2025, Gao W 2025, Lin S 2025, Xue Z 2025, Li X 2023, Zhu XD 2023 (n=6). OS excluding KM-derived: Chi QY 2025, Gao W 2025, Lin S 2025, Xue Z 2025, Yang DL 2025, Li X 2023, Zhu XD 2023, Sun Z 2026 (n=8). PFS/EFS adjusted/TD-Cox: Chi QY 2025, Gao W 2025, Lin S 2025, Li X 2023, Sun Z 2026 (n=5). PFS/EFS excluding KM-derived: same as adjusted/TD-Cox set (n=5).

**Table S11. Data dictionary for extracted variables**

| **Variable** | **Source table** | **Definition** |
| --- | --- | --- |
| Study | HR rows / selected 14 | Author-year study label used throughout the included comparative studies. |
| Record ID | Selected 14 | Stable screening record identifier from the source screening table. |
| Full title | Selected 14 | Article title as recorded in the study extraction table. |
| DOI | Selected 14 | Digital object identifier recorded for the selected study. |
| Starting cohort N | Selected 14 | Number of patients in the conversion-intent starting cohort used for study characterization when recorded. |
| Surgery N | Selected 14 | Number undergoing conversion or salvage hepatectomy/resection. |
| Non-surgery N | Selected 14 | Comparator patients managed without hepatectomy; blank where not reported. |
| endpoint | HR rows | Endpoint as extracted from the report: OS, PFS, or EFS. |
| endpoint_family | HR rows | Analysis family; PFS and EFS were grouped pragmatically as PFS_EFS. |
| HR_CI | HR rows | Hazard ratio and reported or reconstructed 95% confidence interval. |
| Model | HR rows | HR source type, including MVA Cox, TD-Cox, unadjusted Cox, or KM-derived. |
| Quality subgroup | HR rows | Sensitivity grouping based on adjusted/time-dependent versus unadjusted/KM/alternative time origin. |
| Time origin | HR rows | T0=treatment initiation; PC=post-conversion/post-response; UNC=unclear or not fully adjudicable. |
| Adjustment | HR rows | Whether the extracted HR was adjusted, unadjusted, or KM-derived. |
| comparison_type | HR rows | Text description of surgery versus non-surgery contrast used for the HR. |
| endpoint_coherent | HR rows | Whether the endpoint fit the prespecified endpoint family for synthesis. |
| exposure_timing | HR rows | How surgery exposure was defined relative to follow-up and conversion assessment. |
| source_location | HR rows | Page/table/figure location used for HR source verification. |
| E_value | HR rows | Point-estimate E-value for unmeasured confounding calibration. |
| E_value_CI_limit | HR rows | E-value calculated using the confidence bound closest to the null. |
| RR_EU | quantitative bias analysis grid | Association between unfavorable surgical candidacy and surgery selection. |
| RR_UY | quantitative bias analysis grid | Association between unfavorable surgical candidacy and outcome. |
| Bias_factor | quantitative bias analysis grid | Maximum bias factor BF=(RR_EU x RR_UY)/(RR_EU + RR_UY - 1). |
| Timing_bias_factor | Timing scenario | Aggregate timing-bias multiplier exp(lambda_control x months_to_surgery). |

**Table S12. Adapted GRADE certainty-of-evidence assessment**

| **Outcome** | **Starting point** | **Risk of bias** | **Inconsistency** | **Indirectness** | **Imprecision** | **Publication bias** | **Upgrading factors** | **Overall certainty** |
| --- | --- | --- | --- | --- | --- | --- | --- | --- |
| Overall survival | Non-randomized comparative evidence | Downgraded for serious ROBINS-I-informed concerns across endpoint-specific estimates, driven by post-baseline hepatectomy selection, residual surgical-candidacy confounding, participant selection, intervention classification, and timing/immortal-time concerns. | Serious concern: I-squared = 65.7%, prediction interval 0.10-1.04, and variation by model source, time origin, and study design. | Serious concern: the evidence addresses selected post-treatment surgery candidates rather than a randomized causal treatment effect; regimens and time origins were heterogeneous. | Serious concern: the 95% CI excluded the null, but the prediction interval crossed the null and aggregate timing-bias scenarios showed attenuation under plausible assumptions. | Assessed using contour-enhanced funnel plot, SE-based Egger regression, sample-size-based Peters-type regression, and rank-correlation testing. Funnel asymmetry was suggested by Egger and rank-correlation tests, whereas Peters-type regression was not significant; downgraded for possible small-study effects or selective reporting. | Large observed association noted, but not upgraded because residual surgical selection and timing bias could plausibly explain part of the magnitude. | Very low |
| Progression/event-free survival | Non-randomized comparative evidence | Downgraded for serious ROBINS-I-informed concerns across endpoint-specific estimates, driven by post-baseline hepatectomy selection, residual surgical-candidacy confounding, participant selection, intervention classification, and timing/immortal-time concerns. | Serious concern: I-squared = 64.3%, prediction interval 0.16-1.05, and variation by model source, time origin, and study design. | Serious concern: PFS and EFS were grouped as progression/event outcomes, original endpoint definitions varied, and regimens/time origins were heterogeneous. | Serious concern: the 95% CI excluded the null, but the prediction interval crossed the null and aggregate timing-bias scenarios showed attenuation under plausible assumptions. | Assessed descriptively only because the synthesis included 10 estimates and grouped related but non-identical PFS/EFS endpoints. Selective reporting and small-study effects could not be excluded. | Large observed association noted, but not upgraded because residual surgical selection, endpoint variability, and timing bias could plausibly explain part of the magnitude. | Very low |

**Table S13. Comparison with prior systematic reviews and meta-analyses**

| **Reference** | **Scope / aim** | **Included evidence** | **Key pooled findings** | **How the present review differs / adds value** |
| --- | --- | --- | --- | --- |
| Xu H et al., 2024 [5] | Systemic conversion therapies for initially unresectable HCC. | 38 studies, 4042 patients; single-arm and comparative cohorts across TKI, ICI, and locoregional combinations. | Pooled conversion-surgery rate varied by regimen (highest for TKI+ICI+LRT). Converted patients had better OS (HR 0.45, 95% CI 0.35–0.60) and PFS (HR 0.49, 95% CI 0.35–0.70) than non-converted patients. | Focused on regimen-level activity and conversion success. Did not apply quantitative bias analysis or formally evaluate non-randomized comparative HR interpretability. |
| Hu L et al., 2026 [6] | Effectiveness and safety of ICIs in conversion or neoadjuvant therapy for HCC. | 49 studies (36 conversion, n=3497; 13 neoadjuvant, n=569); single-group rate meta-analysis with limited comparative survival data. | Pooled conversion rate 0.23 (95% CI 0.18–0.29), ORR 0.62, DCR 0.87, grade ≥3 AE rate 0.39, resection rate 0.87. Neoadjuvant ICI+surgery vs surgery alone had similar OS/RFS. | Summarized ICI-based strategies and adverse events, but did not quantify selection/timing bias or bound the impact of unmeasured confounding on hepatectomy comparisons. |
| Wang J et al., 2024 [7] | Non-surgery strategy versus hepatectomy in patients with complete response after conversion therapy. | 6 studies, 481 patients with complete response after conversion therapy. | Non-surgery strategy was associated with worse PFS (HR 2.15, 95% CI 1.60–2.90) than hepatectomy; OS did not differ (HR 1.35, 95% CI 0.93–1.96). Outcomes varied by clinical vs radiologic complete response. | Restricted to complete responders and compared surgery vs non-surgery. Did not address the broader initially unresectable population or apply E-values / QBA to survival associations. |
| Present review | Comparative survival outcomes of conversion hepatectomy versus non-hepatectomy management in initially unresectable HCC, with quantitative bias analysis. | 14 comparative survival studies, 24 HR estimates (14 OS, 10 PFS/EFS). | Pooled HR 0.33 (95% CI 0.22–0.48) for OS and 0.41 (95% CI 0.28–0.59) for PFS/EFS. Prediction intervals crossed the null; E-values and timing-bias scenarios indicated sensitivity to residual confounding and timing assumptions. | First review to apply E-values, deterministic and probabilistic quantitative bias analysis, and aggregate timing-bias scenarios specifically to hepatectomy-vs-non-hepatectomy survival comparisons; explicitly framed estimates as associations rather than causal treatment effects. |

*Note: AE, adverse event; DCR, disease control rate; HR, hazard ratio; ICI, immune checkpoint inhibitor; LRT, locoregional therapy; ORR, objective response rate; OS, overall survival; PFS, progression-free survival; QBA, quantitative bias analysis; RFS, recurrence-free survival; TKI, tyrosine kinase inhibitor.*

Table S14. Key full-text reports that appeared potentially eligible but were excluded from quantitative synthesis

| Study/report | Why it appeared potentially eligible | Reason for exclusion from quantitative synthesis |
| --- | --- | --- |
| Zhang X et al. 2026 [4] | Conversion-intent regimen study in initially unresectable hepatocellular carcinoma. | Regimen/conversion-therapy report; no isolated hepatectomy-versus-non-hepatectomy time-to-event HR was available for quantitative synthesis. |
| Zeng ZX et al. 2024 [34] | Conversion-surgery cohort with pathological-response and survival context. | Prognostic/pathological-response analysis among patients undergoing conversion surgery; no eligible non-hepatectomy comparator HR was available. |
| Huang C et al. 2023 [35] | Immunotherapy plus TKI conversion context with radiographic and AFP response endpoints. | Response-prediction study; no eligible hepatectomy-comparison survival HR was available. |

Note: These key full-text reports are provided to clarify PRISMA 2020 item 16b. Complete exclusion categories are summarized in Figure 1 and Methods 2.2.

Table S15. Small-study-effect and reporting-bias diagnostics

| Endpoint | k | Assessment | Result | Interpretation |
| --- | --- | --- | --- | --- |
| OS | 14 | Contour-enhanced funnel plot | Asymmetric, with several smaller estimates in statistically significant protective contours | Suggests possible small-study effects, selective reporting, or true heterogeneity; not definitive proof of publication bias. |
| OS | 14 | SE-based Egger regression | t = -3.71; df = 12; p = 0.003 | Funnel-plot asymmetry detected. |
| OS | 14 | Sample-size-based Peters-type regression | t = 0.77; df = 12; p = 0.456 | No clear sample-size gradient detected by the Peters-type test. |
| OS | 14 | Rank-correlation test | Kendall's tau = -0.47; p = 0.019 | Supports funnel-plot asymmetry. |
| PFS/EFS | 10 | Descriptive funnel-plot diagnostics | Egger p = 0.052; Peters-type p = 0.916; rank-correlation p = 0.073 | Treated as descriptive because k was at the minimum threshold and endpoint labels were grouped. |

Note: Tests were conducted on log-HR estimates. The Peters-type regression used inverse total analytic cohort size as the predictor and is reported as a sample-size-based small-study-effect diagnostic for HR data.

**Appendix S1. R session info, package versions, and reproducibility log**

| **Component** | **Version / value** |
| --- | --- |
| R | R version 4.5.3 (2026-03-11 ucrt) |
| Random seed | 20260504 |
| readr | 2.2.0 |
| dplyr | 1.2.0 |
| tidyr | 1.3.2 |
| stringr | 1.6.0 |
| ggplot2 | 4.0.2 |
| metafor | 4.8.0 |
| meta | 8.3.0 |
| openxlsx | 4.2.8.1 |
| viridis | 0.6.5 |
| ggrepel | 0.9.8 |
| scales | 1.4.0 |
| Analysis code | Archived by the authors. |
| Study source records | Study source records and PRISMA files. |

**Appendix S2. Figure-generation settings and code availability statement**

| **Setting** | **Value** |
| --- | --- |
| Graphics device/export | Dual export to PDF and PNG; PNG resolution 600 dpi; white background. |
| Font/theme | Arial or device-default sans-serif; theme_minimal with bold axis titles and bottom legends. |
| Main figure files | Fig1_PRISMA_flow; Fig2_OS_HR_forest; Fig3_PFS_EFS_HR_forest; Fig4_E_values; Fig5_tipping_point_QBA; Fig6_ROBINSI_domain_summary. |
| Additional file 1 figure files | S1_probabilistic_QBA_OS; S2_probabilistic_QBA_PFS_EFS; S3_aggregate_timing_bias_scenario; S4_endpoint_level_ROBINSI_traffic_light; S5_contour_enhanced_funnel_small_study_effects. |
| Code availability | Figure and analysis code are archived by the authors and are available upon reasonable request. |
| Data availability | Extracted HR rows are recorded in the study extraction table and corresponding HR table. |

**Supplementary figure legends**

Figure S1. Probabilistic QBA distributions for OS. Density curves show simulated bias-adjusted pooled HRs based on the observed OS input (HR_obs = 0.329) under prespecified moderate, strong, and extreme surgical-selection confounding scenarios. The solid vertical line indicates the observed pooled HR, and the dashed vertical line indicates HR = 1.0.

Figure S2. Probabilistic QBA distributions for PFS/EFS. Density curves show simulated bias-adjusted pooled HRs based on the observed PFS/EFS input (HR_obs = 0.408) under prespecified moderate, strong, and extreme surgical-selection confounding scenarios. The solid vertical line indicates the observed pooled HR, and the dashed vertical line indicates HR = 1.0.

Figure S3. Aggregate timing-bias scenario analysis. Scenario-adjusted pooled HRs are shown across assumptions about time from treatment initiation to surgery (2, 4, or 6 months) and comparator median survival (6, 12, 18, or 24 months), using observed inputs of 0.329 for OS and 0.408 for PFS/EFS. This evaluates aggregate sensitivity to timing assumptions.

Figure S4. Endpoint-level ROBINS-I-informed traffic-light plot. Each of the 24 endpoint-specific HR rows is summarized across the adapted domains: residual confounding, participant selection, immortal-time/time-origin concerns, intervention classification, endpoint coherence, and overall risk-of-bias judgement. This endpoint-level plot provides the detailed support for the study-level summary in Figure 6.

Figure S5. Metafor-generated contour-enhanced funnel plots for small-study-effect and reporting-bias assessment. Panel A shows OS estimates, for which k = 14 supported supplementary assessment using funnel-plot diagnostics. Panel B shows PFS/EFS estimates, interpreted descriptively because k = 10 and related but non-identical endpoints were grouped. Plots were generated using the native metafor funnel function. The solid vertical line indicates HR = 1.0, and the dashed blue line indicates the corresponding random-effects pooled estimate. Shaded contours show two-sided statistical-significance regions relative to the null.
